# Targeting Macrophage-to-Myofibroblast Transition Mitigates Progression from Inflammation to Fibrosis in Rosacea

**DOI:** 10.7150/ijbs.128841

**Published:** 2026-02-04

**Authors:** Chengqian Chen, Peiru Wang, Yajing Cao, Dongbin Sung, Yutong Yang, Jin Yang, Jia Liu, Yu Yan, Zhijie Ruan, Jie Dong, Jia Yan, Qihang Chang, Chunying Li, Xiaojing Liu, Xiuli Wang, Qingyu Zeng

**Affiliations:** 1 Institute of Photomedicine, Shanghai Skin Disease Hospital, School of Medicine, Tongji University, Shanghai 200092, China.; 2 Department of Dermatology, Shuguang Hospital Affiliated with Shanghai University of Traditional Chinese Medicine, Shanghai, China.

**Keywords:** rosacea, cutaneous fibrosis, macrophage-to-myofibroblast transition, STAT3 palmitoylation, Bruceine A

## Abstract

Rosacea is a globally prevalent chronic inflammatory skin disorder that markedly impairs quality of life, yet treatment options are limited. A characteristic feature of rosacea is macrophage infiltration, whose role in disease pathogenesis remains incompletely understood beyond inflammation; here, we identify their contribution to fibrotic remodeling through macrophage-to-myofibroblast transition (MMT). Serum proteomics revealed that TGF-β1 was prominently elevated in rosacea patients. Moreover, single-cell RNA sequencing (scRNA-seq), spatial transcriptomics (ST), and histological staining of skin biopsies demonstrated that fibrotic remodeling was already evident at inflammation-dominant stages, with macrophages progressively acquiring myofibroblast-like features through MMT. These observations were recapitulated in LL37-induced mouse models by scRNA-seq and ST, further validated by lineage tracing using *Cx3cr1*-GFP knock-in mice. Interestingly, macrophage depletion markedly alleviated LL37-induced fibrotic remodeling, underscoring the pathogenic role of MMT. Through integrative screening, we subsequently identified Bruceine A (BA), a natural quassinoid that suppressed fibrotic remodeling by reducing MMT and attenuating keratinocyte-driven inflammation *in vivo*. BA directly targeted STAT3 and interfered with its palmitoylation-dependent activation, thereby disrupting profibrotic and inflammatory signaling. Our findings establish MMT as a driver of fibrotic remodeling in rosacea, define STAT3 palmitoylation as a therapeutic target, and position BA as a dual-acting candidate for mechanism-based intervention.

## Introduction

Rosacea is a chronic inflammatory skin disorder with an overall prevalence of approximately 5.1%^1^. It predominantly affects the central face and is clinically classified into erythematotelangiectatic (ETR), papulopustular (PPR), phymatous (PhR), and ocular rosacea (OR), characterized by recurrent erythema, flushing, papules, and pustules, and in advanced PhR, progressing to sebaceous gland hyperplasia and dermal fibrosis^2^. Although not life-threatening, its visible manifestations substantially impair appearance and are associated with a reduced quality of life, higher risks of anxiety and depression, and social stigmatization^3^, ^4^. Current evidence suggests that rosacea pathogenesis is multifactorial, with dysregulated cutaneous immunity constituting the central pathogenic axis, while vascular dysregulation, microbial dysbiosis, environmental triggers, genetic predisposition, and neuroimmune dysregulation have also been reported to contribute^2^, ^5^, ^6^. A well-recognized pathogenic feature of rosacea is the aberrant accumulation of LL37, an antimicrobial peptide that drives keratinocytes to release proinflammatory mediators and perpetuates cutaneous inflammation^7^, ^8^. Thus, LL37 injection constitutes a well-recognized *in vivo* model of rosacea, commonly employed to probe disease mechanisms and evaluate therapeutic candidates^9^-^11^. Despite recent advances, the pathogenic mechanisms of rosacea remain incompletely understood, and targeted therapeutic strategies are still limited.

Substantial evidence indicates that chronic inflammation is a major driver of fibrosis^12^-^14^, which is characterized by overaccumulation of extracellular matrix (ECM) and aberrant tissue remodeling predominantly mediated by activated myofibroblasts that secrete ECM and contract actomyosin-based stress fibers, with transforming growth factor-β1 (TGF-β1) signaling acting as a central regulator^15^, ^16^. Myofibroblasts are traditionally thought to arise from local mesenchymal cells, or through epithelial-mesenchymal transition (EMT) and endothelial-mesenchymal transition (EndoMT)^15^. More recently, macrophage-to-myofibroblast transition (MMT) has been identified as an additional and distinctive source of myofibroblasts^17^-^20^. Cells undergoing MMT are characterized by the co-expression of both macrophage (e.g., CD68 and F4/80) and myofibroblast (α-smooth muscle actin, α-SMA) markers, reflecting their acquisition of profibrotic properties while retaining macrophage lineage traits^21^, ^22^. Based on established criteria from previous studies, this co-expression has been adopted as the definitive signature for identifying MMT cells and has been documented across human tissues, animal models, and *in vitro* systems in studies of heart, lung, kidney, skin, and other fibrotic settings^20^, ^23^-^27^. Such a phenotype positions MMT as a key intermediary that mechanistically links chronic inflammation to fibrotic remodeling. While MMT has been well documented in fibrotic disorders of major organs, its relevance to cutaneous diseases is only beginning to be recognized^26^, ^28^. Notably, rosacea lesions exhibit pronounced macrophage infiltration^7^, ^9^, ^29^, raising the possibility that these cells may extend their pathogenic role beyond inflammation to fibrotic remodeling. Whether macrophages undergo such a transition in rosacea, and whether therapeutic inhibition of this process could ameliorate fibrotic remodeling, however, remains to be determined.

The Janus kinase/signal transducer and activator of transcription (JAK-STAT) pathway is a key signaling cascade that rapidly conveys extracellular signals to the nucleus and thereby controls the transcription of specific genes^30^. Emerging evidence highlights STAT3 as a central regulator of cutaneous inflammation, as demonstrated by studies in psoriasis and atopic dermatitis^31^, ^32^. In rosacea, STAT3 activation has been most notably demonstrated in keratinocytes, where it enhances the expression of proinflammatory cytokines, supports immune infiltration, and contributes to disease progression^33^, ^34^. Recent studies further revealed that STAT3 undergoes S-palmitoylation catalyzed by DHHC7, which promotes its membrane recruitment and subsequent phosphorylation by JAK2, thereby implicating palmitoylation as a potential regulator of STAT3-driven inflammation and fibrosis^35^, ^36^. Although the TGF-β1/Smad3 pathway has been established as a major driver of MMT, the JAK-STAT pathway may also play a role in this process^19^, ^22^, ^37^, ^38^. Yet, whether STAT3 directly contributes to MMT in rosacea remains to be elucidated.

In this study, we delineated a previously underappreciated connection between fibrosis and rosacea by showing that TGF-β1 is prominently elevated in rosacea. Using both scRNA-seq and ST, we observed that fibrotic remodeling was already evident even at inflammation-dominant stages, during which macrophages progressively acquired myofibroblast-like properties and transitioned toward a fibrogenic state in the skin of rosacea patients and LL37-induced mouse models. This finding was substantiated by multiplexed immunohistochemistry (mIHC) staining of human and mouse skin biopsies, and was further corroborated by lineage tracing in *Cx3cr1*-GFP knock-in mice. Through integrative screening, we identified BA, a quassinoid naturally present in *Brucea javanica*, as a therapeutic candidate with potent anti-fibrotic and anti-inflammatory activities. BA treatment attenuated MMT in the dermis, thereby alleviating fibrotic remodeling, and concurrently suppressed keratinocyte-mediated inflammatory pathways in the epidermis. Mechanistically, prolonged LL37 stimulation drove STAT3 palmitoylation and subsequent phosphorylation-dependent nuclear translocation, which served as a pivotal trigger for MMT and activation of keratinocyte inflammatory cascades. BA specifically inhibited STAT3 palmitoylation and thereby prevented its downstream activation, severing the inflammatory-fibrotic axis in rosacea. In summary, our study identifies MMT as a therapeutic target in rosacea and demonstrates that its inhibition by BA provides a feasible strategy to intercept the progression from inflammation to fibrosis.

## Methods

### Human samples

Skin biopsies and serum samples were obtained from patients with rosacea and healthy volunteers at Shanghai Skin Disease Hospital. Patients were diagnosed by clinical and histopathological examination. Biopsies were collected from the central face, including lesional skin from patients and normal skin from controls. Serum was collected by venipuncture, processed, and stored at -80 °C until analysis. Written informed consent was obtained from all participants. All studies involving human tissues were conducted in accordance with the Declaration of Helsinki and with the approval of the Research Ethics Committee of Shanghai Skin Disease Hospital (2025-13).

### Animals

BALB/c mice were obtained from the Shanghai Laboratory Animal Center (Shanghai, China), while *Cx3cr1*-GFP knock-in mice (C57BL/6 background) were obtained from Cyagen Biosciences (Taicang, Jiangsu, China). Eight-week-old female mice were used for experiments. Mice were kept under specific pathogen-free conditions with a 12-h light/dark cycle, had free access to standard chow and water, and were randomly assigned to experimental groups. All animal experiments were conducted in accordance with the ethical standards set by China's Institutional Animal Care and Use Committee at Tongji University (SYXK (hu) 2020-0002) and with the approval of the Animal Ethics Committee of Shanghai Skin Disease Hospital (2024-41).

### Mouse model of rosacea and treatments

Mice had the dorsal skin shaved one day before the first LL37 peptide (MedChemExpress, Monmouth Junction, NJ, USA) injection. For the short-term model, intradermal injection of LL37 (40 μL, 320 μM) was performed every 12 h for 2 consecutive days. For the long-term model, mice received intradermal injections of LL37 (40 μL, 320 μM) once daily for 20 consecutive days. Twenty-four hours after the last LL37 injection, the gross cutaneous phenotype was documented by photography, and mice were sacrificed to collect dorsal skin tissues for subsequent analyses.

For therapeutic intervention, BA (0.5 or 2 mg/kg; MedChemExpress) was administered by intraperitoneal injection simultaneously with the second LL37 injection, and subsequently administered once every two LL37 injections. In the short-term model, dexamethasone (DEX, 10 mg/kg; Yeasen, Shanghai, China) served as a positive control, while colivelin (CLN, 1 mg/kg; MedChemExpress) was used as a pharmacological activator of STAT3; both were administered intraperitoneally on the same schedule.

For macrophage depletion, mice were subjected to daily LL37 administration for 7 consecutive days. Clodronate-containing liposomes (CL; Yeasen) were administered intraperitoneally, with an initial dose of 200 μL given 2 days before the first LL37 injection (corresponding to a body weight of approximately 20 g), followed by maintenance injections of 100 μL every other day to prevent macrophage repopulation.

### Cell culture and treatment

THP-1 cells were maintained in RPMI-1640 medium supplemented with 10% fetal bovine serum and 1% penicillin/streptomycin solution (Thermo Fisher Scientific, Waltham, MA, USA) at 37 °C in a humidified incubator containing 5% CO_2_. Cells were seeded into six-well plates at a density of 2 × 10⁶ per well and exposed to 100 ng/mL phorbol 12-myristate 13-acetate (PMA; MedChemExpress) for 24 h to induce differentiation into resting (M0) macrophages. For subsequent experiments, M0 macrophages were pretreated with SIS3 (10 μM, 1 h; MedChemExpress), Stattic (10 μM, 1 h; MedChemExpress), BA (2.4 μM, 6 h), or 2-bromopalmitate (2-BP, 40 μM, 6 h; MedChemExpress), with vehicle-treated cells serving as controls, followed by stimulation with LL37 (4 μM) for 48 h.

Human dermal fibroblast (HDF) cells and HaCaT cells were cultured in Dulbecco's Modified Eagle Medium (DMEM) supplemented with 10% fetal bovine serum and 1% penicillin/streptomycin solution under the same culture conditions. For HaCaT cell-based experiments, cells were pretreated with either BA (2.4 μM), CLN (50 μg/mL), Stattic (10 μM), or vehicle for 1 h, followed by stimulation with LL37 (12 μM) for 6 h.

### Cell coculture system

For coculture, macrophages and fibroblasts were separated by transwell inserts (Corning, NY, USA) with a pore size of 0.4 μm. In brief, THP-1-derived macrophages were seeded onto the membrane of the transwell inserts and subjected to the indicated treatments, while HDF cells were seeded in the bottom of the plate wells. The inserts were then placed into the wells, and coculture was maintained for 48 h.

### Single-cell RNA sequencing

Under sterile conditions, freshly collected human skin tissues (4 controls, 2 ETR, 2 PPR) and mouse skin tissues (3 controls, 3 short-term LL37-treated) were washed twice with pre-cooled RPMI-1640 containing 0.04% BSA, minced (~0.5 mm³), digested at 37 °C for 30-60 min in RPMI-1640 with 0.04% BSA and 0.2% collagenase II, filtered through a 40 μm strainer, subjected to red blood cell lysis, washed, and resuspended. Single-cell suspensions were adjusted to 700-1200 cells/μL. Libraries were prepared with the MobiCube High-throughput Single Cell 3' Transcriptome Set V2.1 (Cat# PN-S050200301) and sequenced on the Illumina Nova 6000 platform in PE150 mode. The FASTQ files were processed and aligned to the GRCh38 (human) and GRCm39 (mouse) reference genomes using MobiVision software (v3.2), with unique molecular identifier (UMI) counts summarized for each barcode. Analysis of the filtered UMI count matrix was performed using the Seurat (v4.0.0) R package. Low-quality cells and potential multiplets (DoubletFinder, v2.0.3) were excluded. Principal component analysis (PCA) was conducted for dimensionality reduction, and cells were clustered using a graph-based approach and visualized with Uniform Manifold Approximation and Projection (UMAP). Differential expression was assessed using FindMarkers (test.use = presto), and differentially expressed genes (DEGs) were defined as those with P < 0.05 and |log2 fold change| > 0.58. Sequencing and bioinformatics analyses were supported by OE Biotech Co., Ltd. (Shanghai, China).

### Spatial transcriptome sequencing

Freshly collected human and mouse skin tissues were cut into appropriately sized blocks, immediately fixed in 4% PFA, and paraffin-embedded. FFPE sections (5 μm) were mounted on anti-slide slides according to the 10x Genomics protocol. Deparaffinization, H&E staining, imaging, and decrosslinking were performed following the 10x Genomics protocol (CG000520). Spatial transcriptomic libraries were generated using the Visium CytAssist Spatial Gene Expression for FFPE kit (10x Genomics; PN-1000520 for Human, 6.5 mm; PN-1000521 for Mouse, 6.5 mm), with probe hybridization, probe release, and library construction performed according to the manufacturer's protocol (CG000495), and sequenced on the BGI DNBSEQ-T7 sequencing platform in PE100 mode. The FASTQ files were preprocessed and mapped to the GRCh38 (human) and GRCm39 (mouse) reference genomes through Space Ranger (v2.0.1, 10x Genomics), and UMI counts were aggregated per barcode. Spots overlapping with tissue regions were distinguished from the background using image-based analysis. The filtered UMI count matrix was subsequently analyzed with the Seurat R package (v4.1.0). To identify DEGs, the FindMarkers function in Seurat (test.use = presto) was applied. Genes with P < 0.05 and |log2 fold change| > 0.58 were considered significantly differentially expressed. Sequencing and bioinformatics analyses were supported by OE Biotech Co., Ltd.

### Bulk RNA sequencing

For RNA-seq and DEG identification, libraries were sequenced using the Illumina NovaSeq 6000 platform, producing 150 bp paired-end reads. Raw FASTQ files were quality-filtered with fastp to obtain clean reads, which were then mapped to the reference genome using HISAT2. FPKM was calculated for each gene, and read counts were derived with HTSeq-count. In R (v3.2.0), PCA was applied to examine biological replication among samples. Differential expression was analyzed using DESeq2, with DEGs identified when q < 0.05 and |log2FoldChange| > 1. Hierarchical clustering of DEGs was performed in R to characterize expression patterns across different groups and samples. Gene Ontology (GO) and Kyoto Encyclopedia of Genes and Genomes (KEGG) enrichment analyses were conducted using the hypergeometric distribution, and summary plots were generated in R. Gene Set Enrichment Analysis (GSEA) was performed with the GSEA software to assess whether predefined gene sets were significantly enriched at the top or bottom of the ranked gene list.

### Histological staining

Mouse skin and major organs were processed to obtain paraffin-embedded sections (4 μm thick), which were stained with hematoxylin and eosin (H&E). In addition, skin sections were further subjected to Masson's trichrome, Sirius Red, and Toluidine Blue staining according to standard protocols.

Immunohistochemistry (IHC) staining was performed on mouse and human skin tissues. Specifically, mouse skin sections were incubated with primary antibodies against α-SMA (1:200; Cat# abs130621, Absin, Shanghai, China), vimentin (1:200; Cat# D21H3, Cell Signaling Technology, Danvers, MA, USA), and CD31 (1:1000; Cat# ab281583, Abcam, Cambridge, UK), while human skin sections were incubated with anti-STAT3 antibody (1:200; Cat# A19566, ABclonal, Wuhan, China). Sections were incubated with HRP-conjugated secondary antibodies once the primary antibodies had been removed. Subsequently, the peroxidase substrate 3,3'-diaminobenzidine (DAB; Servicebio, Wuhan, China) was applied, and hematoxylin was used for counterstaining.

Additionally, mIHC staining was performed on human and mouse skin tissues. Briefly, sections were stained with standard primary antibodies and paired with the TSA kit, followed by 4',6-diamidino-2-phenylindole (DAPI) staining. Specifically, human skin sections were incubated with antibodies against CD68 (1:200; Cat# BX50031, Biolynx, Hangzhou, China) and α-SMA (1:400; Cat# ab124964, Abcam), while mouse skin sections were incubated with antibodies against F4/80 (1:500; Cat# GB113373, Servicebio) and α-SMA (1:200). To evaluate the efficiency of CL in macrophage depletion, mouse skin sections were stained using an anti-F4/80 antibody (1:500).

For immunofluorescence (IF) staining in lineage-tracing experiments, frozen mouse skin sections were incubated with anti-α-SMA antibody (1:2000; Cat# ab124964, Abcam), and GFP signals were directly detected at an excitation wavelength of 488 nm. For assessment of STAT3 nuclear translocation, macrophages and keratinocytes cultured on glass coverslips were subjected to IF staining with an anti-STAT3 antibody (1:100). Nuclei were uniformly counterstained with DAPI. Cultured cells on glass coverslips were imaged using a confocal microscope (Nikon, Tokyo, Japan), while all other stained tissue slides were scanned with a Pannoramic MIDI digital scanner (3DHISTECH, Budapest, Hungary). All images were subsequently analyzed with CaseViewer 2.4 and ImageJ software.

### EdU incorporation assay

HDF proliferation in the coculture system was evaluated using a 5-ethynyl-2'-deoxyuridine (EdU) incorporation assay according to the manufacturer's instructions (CellorLab, Shanghai, China). Briefly, cells were incubated with EdU (10 μM) for 2 h, followed by fixation with 4% paraformaldehyde and permeabilization with 0.3% Triton X-100. Incorporated EdU was detected via click chemistry, and nuclei were counterstained with Hoechst 33342. Fluorescence images were acquired using a fluorescence microscope, and the proportion of EdU⁺ cells was quantified.

### Enzyme-Linked Immunosorbent assay (ELISA)

Culture supernatants from macrophages were quantified for TGF-β1 levels using commercial human ELISA kits (YOBIBIO, Shanghai, China) in accordance with the manufacturer's guidelines. Mouse skin tissues were analyzed for TGF-β1 levels using commercial ELISA kits (Meilunbio, Dalian, China), with results normalized to protein concentration.

### Multiplex secretome analysis

Supernatants from tissue homogenates were collected after treatment. The fluorescent coded microspheres (XMplex 4-Plex Custom Panel, XM-BIOTECH, Wuhan, China) were mixed and supernatants were added in accordance with the manufacturer's instructions with support from XM-BIOTECH.

### Western blotting (WB)

Western blotting assays were performed as previously described^39^. For separation of nuclear and cytoplasmic proteins in macrophages, a nuclear and cytoplasmic protein extraction kit (Beyotime, Shanghai, China) was used following the manufacturer's instructions. Primary antibodies used were GAPDH (1:2000; Cat# AF7021, Affinity, Cincinnati, OH, USA), α-SMA (1:1000; Cat# abs130621, Absin), Collagen I (1:1000; Cat# ab316222, Abcam), STAT3 (1:2000; Cat# A19566, ABclonal), p-STAT3 (Tyr705) (1:1000; Cat# 9131, Cell Signaling Technology), and Histone H3 (1:1000; Cat# AF0863, Affinity).

### Quantitative real-time PCR (qPCR)

Total RNA was extracted from cultured cells using TRIzol reagent (Thermo Fisher Scientific) according to the manufacturer's instructions. First-strand cDNA was synthesized with the Hifair®II 1st Strand cDNA Synthesis SuperMix (Yeasen). Quantitative PCR was conducted on a LightCycler® 480 Instrument II (Roche, Basel, Switzerland) using a standard SYBR Green PCR kit (Yeasen). Relative gene expression levels were calculated using the 2^-ΔΔCt^ method. Primer sequences are provided in Table S1.

### Network pharmacology

To identify candidate therapeutic agents, a database-driven screening strategy was employed. Entries annotated to target fibrosis or inflammation were first retrieved from the ITCM database (http://itcm.biotcm.net/). In parallel, the TCMSP database (http://tcmspw.com/tcmsp.php) was queried for candidates targeting all core pathogenic factors in fibrosis (*IL6*, *IL10*, *TGFB1*, *TNF*)^40^, and the HIT database (http://hit2.badd-cao.net/) was queried for candidates targeting all core pathogenic factors in rosacea (*IFNG*, *TNF*, *IL6*, *IL10*, *IL17A*, *AQP3*, *MMP9*, *TLR2*, *TRPV1*, VEGFA)^41^. The final set of candidate therapeutic agents was defined as the intersection of these four collections.

To investigate the potential mechanisms of BA, putative targets were first predicted using PharmMapper (http://www.lilab-ecust.cn/pharmmapper/) and SuperPred (http://prediction.charite.de/). In parallel, rosacea-related targets were retrieved from the GEO dataset GSE65914 (https://www.ncbi.nlm.nih.gov/geo/). All predicted drug targets and disease-associated targets were standardized to gene symbols using the UniProt database (https://www.uniprot.org/). Overlapping targets were then identified using Venny 2.1.0 (https://bioinfogp.cnb.csic.es/tools/venny/index.html). The common targets were subsequently uploaded to the STRING database (https://cn.string-db.org/), with the species restricted to Homo sapiens, to generate a protein-protein interaction (PPI) network. The network was then visualized using Cytoscape 3.7.2, and a core subnetwork was reconstructed to highlight key interactions.

### Molecular docking

The structure of BA was obtained from the PubChem database (CID: 160006, https://pubchem.ncbi.nlm.nih.gov/). The crystal structures of human JAK1 (PDB ID: 3EYG), JAK2 (PDB ID: 2B7A), TYK2 (PDB ID: 3LXN), and STAT3 (PDB ID: 6NJS) were downloaded from the RCSB PDB database (https://www.rcsb.org/). Protein structures were preprocessed using the Protein Preparation Wizard in Schrödinger Maestro 12.8, including hydrogen addition, water removal, completion of missing atoms, and energy optimization with the OPLS2005 force field. The binding pockets were defined based on co-crystallized ligands or reported inhibitor-binding sites. BA was prepared using the LigPrep module by hydrogenation and energy optimization. Molecular docking was conducted using the Ligand Docking module in XP mode, and binding interactions were visualized with PyMol.

### Cellular thermal shift assay (CETSA)

Cells were treated with 2.4 μM BA or DMSO for 2 h. The resulting cell suspension was evenly divided into aliquots, each heated at a distinct temperature for the same duration, followed by snap-freezing in liquid nitrogen. After three freeze-thaw cycles and centrifugation, the supernatants were collected for western blotting.

### Acyl-biotin exchange (ABE)

For detection of palmitoylation, STAT3 was immunoprecipitated (IP) from macrophages subjected to different treatments using an immunoprecipitation kit with Protein A+G Magnetic Beads (Beyotime) and an anti-STAT3 antibody. The immunoprecipitated material was then subjected to ABE using a commercial IP-ABE palmitoylation kit (AIMSMASS, Shanghai, China) according to the manufacturer's instructions. In brief, free thiols were initially blocked, followed by selective cleavage of palmitoyl thioester bonds with or without hydroxylamine (NH_2_OH). The newly exposed cysteines were then labeled with biotin, and proteins were subsequently eluted and resolved by SDS-PAGE. Biotinylated STAT3 was detected by western blotting using HRP-conjugated streptavidin.

### Quantification and statistical analysis

Statistical analyses were conducted using GraphPad Prism (v10.0). Quantitative results are expressed as the mean ± standard deviation (SD). Comparisons between two groups were assessed using an unpaired Student's t-test, with Welch's correction applied in cases where the assumption of homogeneity of variances was violated. When group comparisons involved three or more conditions, statistical differences were assessed using one-way or two-way ANOVA, and pairwise testing was adjusted with Bonferroni correction. Graphical representations depict either representative or pooled data from independent replicate experiments.

## Results

### MMT is enhanced in rosacea, contributing to fibrotic remodeling

**Figure 1:**
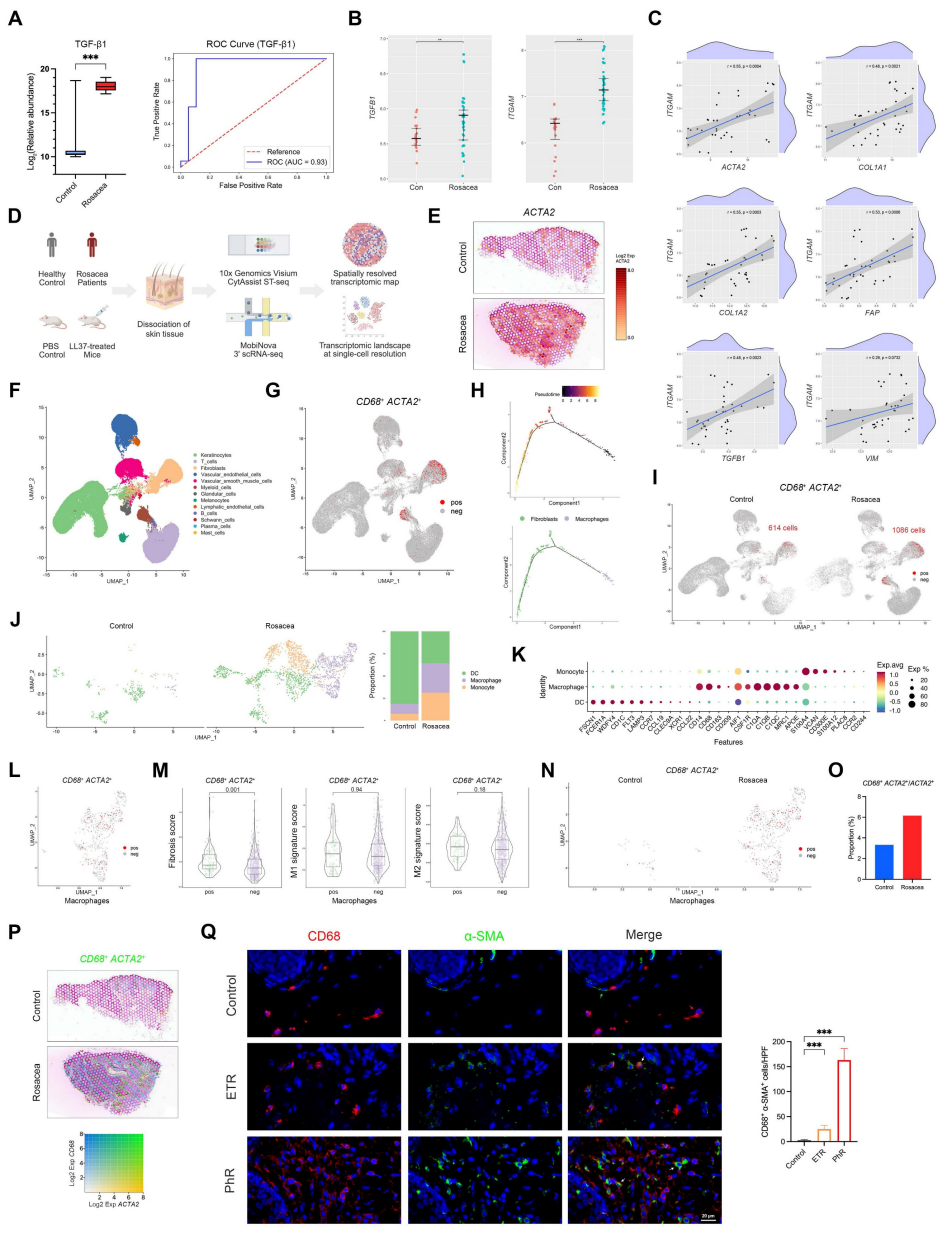
Enhanced MMT underlies fibrotic remodeling in rosacea patients. (A) Serum proteomics comparing rosacea patients (n = 18) and healthy controls (n = 19). Box plot of relative abundance of TGF-β1 (left). ROC curve based on serum TGF-β1 level with AUC (right). (B, C) Relative expression levels (B) and correlation analysis (C) from GEO dataset GSE65914. (D) Schematic diagram of the experimental workflow with human and mouse ST and scRNA-seq (created with BioRender.com). (E) Spatial feature plots of *ACTA2*. (F) UMAP plot showing clusters of major skin cell populations from rosacea patients (n = 4) and healthy controls (n = 4). (G) Feature plot highlighting *CD68*⁺ *ACTA2*⁺ cells. (H) Pseudotime trajectory of *CD68*⁺ *ACTA2*⁺ cells from macrophages to fibroblasts. (I) Feature plot of *CD68*⁺ *ACTA2*⁺ cells, separated by the two groups. (J, K) UMAP subclustering of myeloid cells in the two groups with proportions of each subset (J), and representative marker genes by dot plot (K). (L) Feature plot of *CD68*⁺ *ACTA2*⁺ cells within the macrophage subcluster. (M) Fibrosis, M1, and M2 signature scores of *CD68*⁺ *ACTA2*⁺ macrophages versus other macrophages. (N) Feature plot of *CD68*⁺ *ACTA2*⁺ macrophages, separated by the two groups. (O) Proportions of *CD68*⁺ *ACTA2*⁺ cells among *ACTA2*⁺ cells. (P) Spatial feature plots of co-expression of *CD68* and *ACTA2*. (Q) Representative mIHC images of CD68 (red), α-SMA (green) and DAPI (blue) in control, ETR and PhR skin, with quantification of CD68⁺ α-SMA⁺ cells (n ≥ 3). White arrows, CD68⁺ α-SMA⁺ cells; red arrows, CD68⁺ cells; green arrows, α-SMA⁺ cells. Data are presented as mean ± SD; ^*^P < 0.05, ^**^P < 0.01, ^***^P < 0.001; ns, not significant. Scale bar = 20 μm.

We performed proteomic analyses on serum samples from patients with rosacea (n = 18) and healthy controls (n = 19), with detailed patient characteristics and demographics provided in Table S2. This analysis revealed marked alterations in protein expression in rosacea patients, among which TGF-β1 exhibited one of the most pronounced fold changes (Fig. S1A and Fig. 1A, left panel). Receiver operating characteristic (ROC) curve analysis further demonstrated that TGF-β1 effectively discriminated patients with rosacea from healthy controls, with an area under the curve (AUC) of 0.93 (Fig. 1A, right panel). These results suggest a potential involvement of fibrotic remodeling in the pathogenesis of rosacea.

Previous studies have consistently reported a marked increase in dermal macrophage infiltration in rosacea lesions^7^, ^9^, ^29^. In parallel, accumulating evidence from other fibrotic disorders suggests that macrophages can promote disease progression through MMT^17^-^20^. To investigate whether a similar mechanism operates in rosacea skin, we first analyzed external GEO datasets and observed elevated expression of *TGFB1, ITGAM, CD14, CX3CR1,* and *MRC1* in lesional skin of rosacea patients (Fig. 1B and Fig. S1B). Correlation analysis further revealed that myeloid cell signatures, particularly *MRC1*-defined M2 macrophages, were strongly and positively associated with profibrotic markers including *ACTA2, COL1A1, COL1A2, FAP, TGFB1,* and *VIM* (Fig. 1C and Fig. S1C). To validate these findings, we performed ST profiling of skin biopsies from patients and healthy controls, which confirmed that fibrosis- and macrophage-associated genes were markedly enriched in the dermis of rosacea lesions (Fig. 1D-E and Fig. S1D-S1E).

Based on these results, we next investigated whether MMT is present in human skin, particularly in rosacea. To this end, we performed scRNA-seq on skin biopsies from four patients with rosacea and four healthy controls (Fig. 1D). After quality control and filtering, we retained 37,650 single-cell transcriptomes from rosacea lesions and 36,199 from healthy controls. Subsequent dimensionality reduction and clustering by UMAP revealed multiple transcriptionally distinct cell clusters. These clusters were annotated as major skin cell populations based on canonical marker expression, including keratinocytes, fibroblasts, T cells, B cells, plasma cells, myeloid cells, vascular endothelial cells, vascular smooth muscle cells, lymphatic endothelial cells, glandular cells, melanocytes, Schwann cells, and mast cells (Fig. 1F and Fig. S1F). Because macrophages undergoing MMT are characterized by the co-expression of macrophage and myofibroblast markers, we examined, in accordance with established criteria from previous studies, the presence of cells co-expressing *CD68* and *ACTA2* at the single-cell level. Indeed, a subset of *CD68*⁺ *ACTA2*⁺ cells was detected, mainly localized within the myeloid and fibroblast clusters on the UMAP plot (Fig. 1G). We further subjected these double-positive cells to pseudotime trajectory analysis, which revealed a continuous developmental path from macrophage-like states toward fibroblast-like states (Fig. 1H). These results suggest that a subset of macrophages may acquire myofibroblast features, thereby providing evidence for the presence of MMT in human skin.

We next examined whether MMT is enhanced in rosacea. Direct comparison of *CD68*⁺ *ACTA2*⁺ cells revealed a striking increase in rosacea lesions (1,086 cells) compared with healthy skin (614 cells) (Fig. 1I). To clarify the cellular source of this increase, we subclustered the myeloid compartment and found a marked expansion of macrophages in rosacea, consistent with previous reports (Fig. 1J-K). As expected, *CD68*⁺ *ACTA2*⁺ macrophages were observed within the macrophage population (Fig. 1L). Gene set scoring showed that double-positive macrophages had a significantly higher fibrosis score than other macrophages, whereas the M2 signature score was also elevated but did not reach statistical significance (Fig. 1M). Importantly, rosacea lesions harbored not only more double-positive macrophages overall (Fig. 1N), but also a higher proportion of *ACTA2*⁺ cells that originated from macrophages (Fig. 1O). To validate these findings, we examined the spatial distribution of *CD68* and *ACTA2* expression using ST. Consistent with the single-cell analysis, rosacea lesions showed a greater number of regions with co-expression of *CD68* and *ACTA2* compared with healthy skin (Fig. 1P). Moreover, mIHC staining confirmed the presence of CD68⁺ α-SMA⁺ macrophages, which were markedly increased in ETR lesions and further expanded in PhR lesions compared with healthy controls (Fig. 1Q and Fig. S2A). Together, these results provide convergent evidence that MMT is a defining feature of rosacea lesions, a process that is already increased in inflammation-predominant subtypes and becomes particularly pronounced in PhR, concomitant with the progression of fibrotic remodeling.

### Macrophages transdifferentiate into α-SMA⁺ myofibroblasts *in vivo*, progressively expanding with sustained LL37 stimulation and concomitant fibrotic remodeling

**Figure 2:**
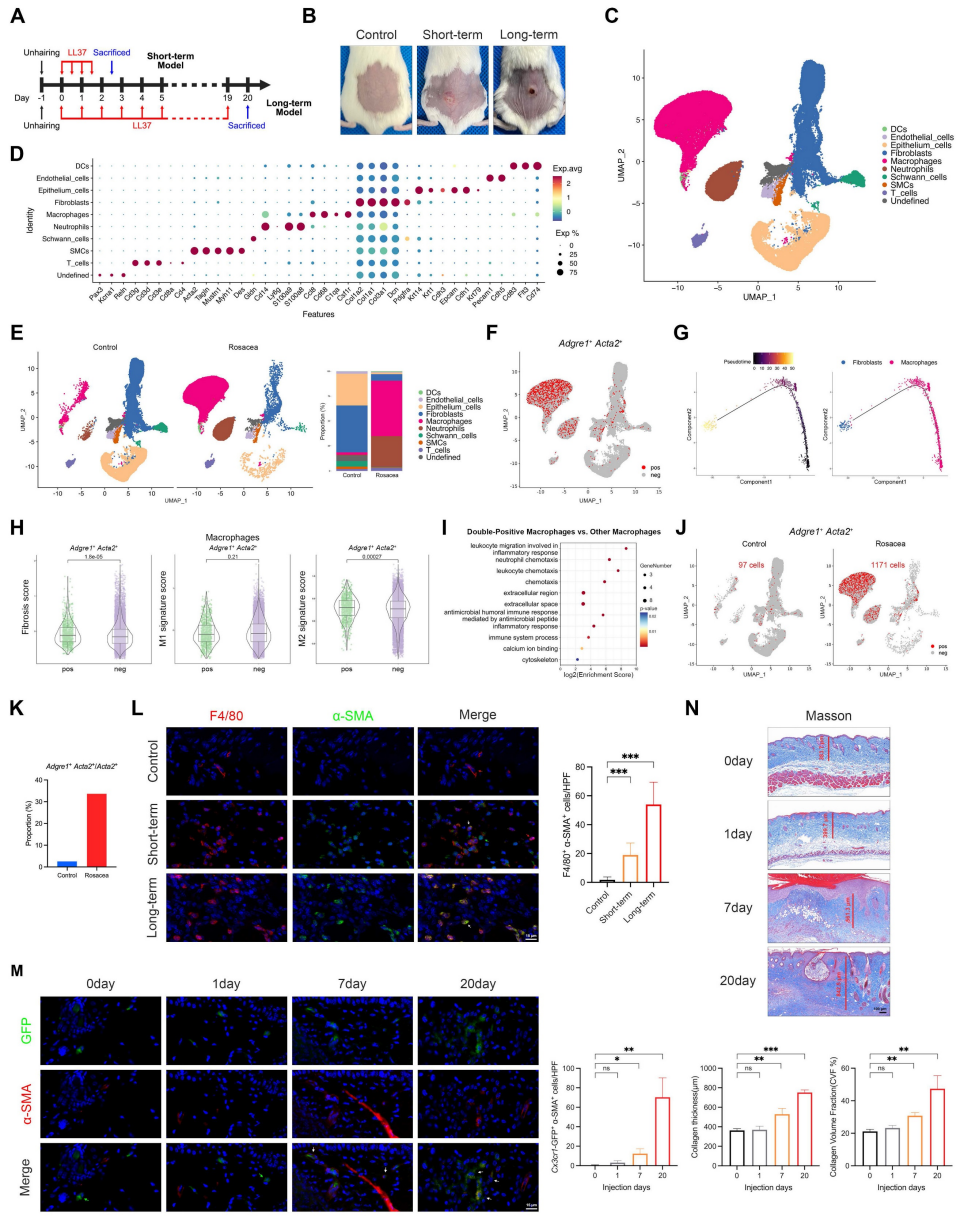
MMT progressively expands *in vivo* with sustained LL37 stimulation, accompanied by fibrotic remodeling. (A) Schematic diagram of short- and long-term LL37-induced mouse models. (B) Representative skin manifestations. (C) UMAP plot showing clusters of major skin cell populations from short-term rosacea-like dermatitis model (n = 3) and controls (n = 3). (D) Dot plot showing representative marker genes for each cell type. (E) UMAP plots separated by control and rosacea-like dermatitis model, with proportions of each cell population. (F) Feature plot highlighting *Adgre1*⁺ *Acta2*⁺ cells. (G) Pseudotime trajectory of *Adgre1*⁺ *Acta2*⁺ cells from macrophages to fibroblasts. (H, I) Fibrosis, M1, and M2 signature scores (H) and KEGG pathway enrichment analysis (I) of *Adgre1*⁺ *Acta2*⁺ macrophages versus other macrophages. (J) Feature plot of *Adgre1*⁺ *Acta2*⁺ cells, separated by the two groups. (K) Proportions of *Adgre1*⁺ *Acta2*⁺ cells among *Acta2*⁺ cells. (L) Representative mIHC images of F4/80 (red), α-SMA (green) and DAPI (blue) in control, short-term and long-term models, with quantification of F4/80⁺ α-SMA⁺ cells (n = 6). White arrows, F4/80⁺ α-SMA⁺ cells; red arrows, F4/80⁺ cells; green arrows, α-SMA⁺ cells. (M) Representative IF images of GFP (green), α-SMA (red) and DAPI (blue) from *Cx3cr1*-GFP knock-in mice subjected to LL37 stimulation for different durations, with quantification of *Cx3cr1*-GFP⁺ α-SMA⁺ cells (n = 3). White arrows, GFP⁺ α-SMA⁺ cells; green arrows, GFP⁺ cells. (N) Representative Masson's trichrome staining at different durations of LL37 stimulation, with quantification of collagen thickness and collagen volume fraction (CVF%) (n = 3). Data are presented as mean ± SD; *P < 0.05, **P < 0.01, ***P < 0.001; ns, not significant. Scale bars: 15 μm (L, M), 100 μm (N).

Given the enhanced MMT observed in human rosacea, we further employed LL37-induced mouse models to determine whether this process is recapitulated *in vivo*. We first established a short-term rosacea-like dermatitis model by intradermally injecting LL37 into the dorsal skin of BALB/c mice for 2 consecutive days (Fig. 2A-B). To characterize cellular alterations within this model, we performed scRNA-seq of LL37-treated and control mice. After quality control, we retained 28,814 and 33,152 single-cell transcriptomes from the LL37-treated and control groups, respectively. UMAP resolved discrete clusters that were annotated as epithelium cells, fibroblasts, endothelial cells, smooth muscle cells (SMCs), macrophages, dendritic cells (DCs), T cells, neutrophils, Schwann cells, and an undefined cluster (Fig. 2C-D).

Consistent with human rosacea, the mouse model exhibited parallel cellular and molecular features of MMT. Specifically, macrophages were markedly expanded compared with controls (Fig. 2E). *Adgre1*⁺ *Acta2*⁺ cells were identified, indicating the presence of MMT *in vivo* (Fig. 2F). Pseudotime trajectory analysis also revealed a transition from macrophage-like to fibroblast-like states (Fig. 2G). Functionally, double-positive macrophages displayed significantly higher fibrosis and M2 signature scores than other macrophages (Fig. 2H), and their upregulated genes were enriched in inflammation- and fibrosis-related pathways (Fig. 2I). Moreover, LL37-treated mice harbored substantially more *Adgre1*⁺ *Acta2*⁺ cells than controls (Fig. 2J), together with a markedly higher proportion of *Acta2*⁺ cells originating from macrophages (Fig. 2K). Analysis with mIHC confirmed an increase in F4/80⁺ α-SMA⁺ macrophages in the short-term LL37-induced model (Fig. 2L and Fig. S2B). To further investigate the contribution of MMT to fibrotic remodeling, we established a long-term LL37-induced model (20 days, Fig. 2A), which exhibited a much more extensive accumulation of double-positive macrophages compared with the short-term model (Fig. 2L and Fig. S2B).

To further elucidate the cellular origin of MMT in rosacea, we utilized *Cx3cr1*-GFP knock-in mice, which enable specific labeling and lineage tracing of macrophage-derived populations under sustained LL37 stimulation for 20 days. IF analysis revealed that a subset of *Cx3cr1*-GFP⁺ cells co-expressed α-SMA, providing direct evidence that myofibroblast-like cells can arise from macrophages *in vivo* (Fig. 2M and Fig. S2C). Quantitative analyses demonstrated a progressive increase in *Cx3cr1*-GFP⁺ α-SMA⁺ cells over time, with the most prominent accumulation observed at day 20 (Fig. 2M). In parallel, Masson's trichrome staining revealed increased dermal collagen thickness along with more densely packed collagen fibers, indicating that the expansion of macrophage-derived α-SMA⁺ myofibroblasts is closely associated with fibrotic remodeling (Fig. 2N). Altogether, these results establish that macrophages can transdifferentiate into α-SMA⁺ myofibroblasts *in vivo*, a process that emerges at early stages and progressively intensifies with sustained LL37 stimulation, concomitantly with the progression of fibrotic remodeling.

### LL37 directly drives MMT and reveals macrophages as key amplifiers of fibrotic remodeling

**Figure 3:**
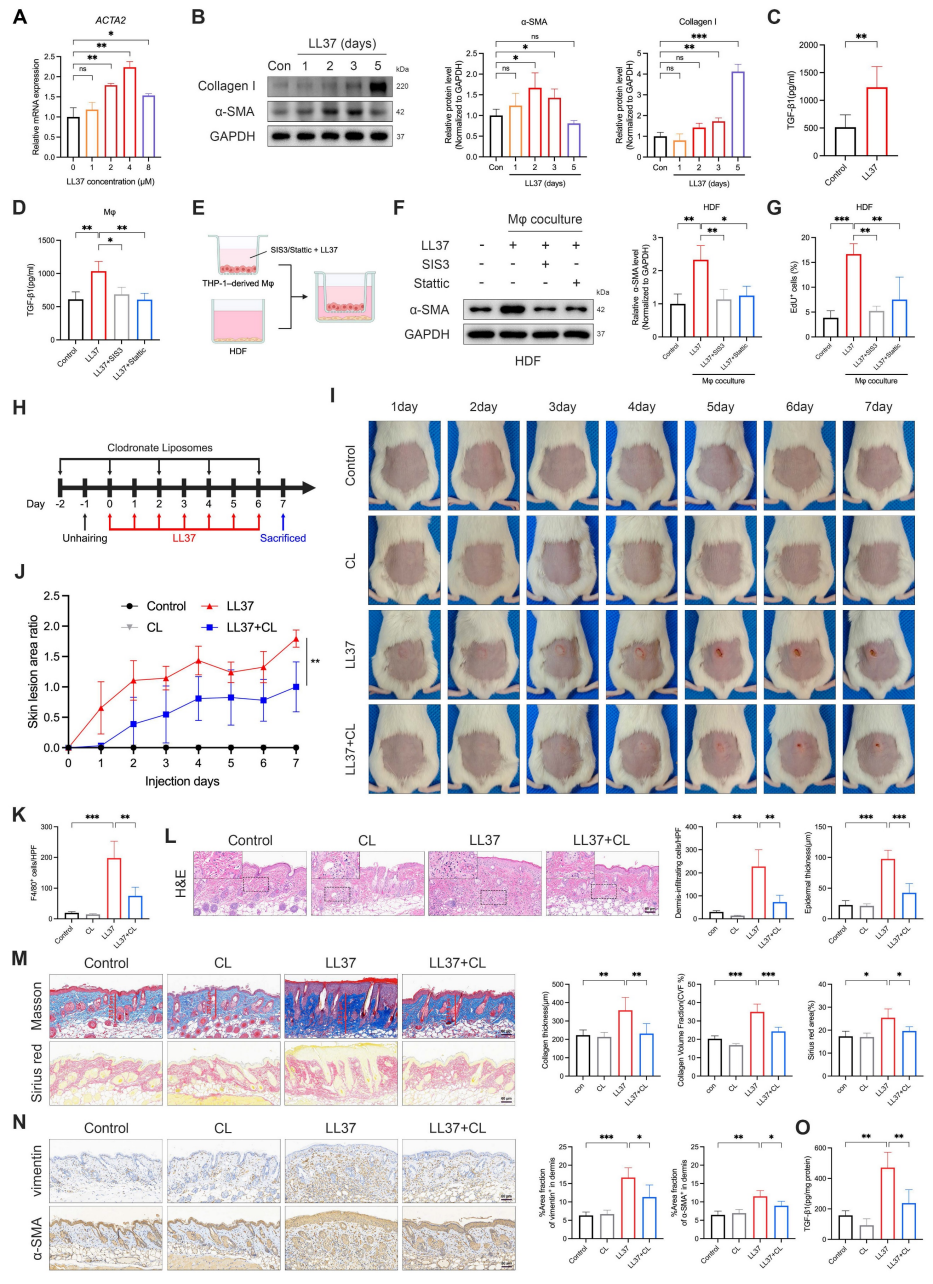
LL37-driven MMT contributes to fibrosis, while macrophage depletion attenuates inflammatory infiltration and fibrotic remodeling. (A) Relative mRNA expression of *ACTA2* in THP-1-derived macrophages treated with different concentrations of LL37. (B) WB analysis of α-SMA and collagen I after LL37 stimulation for the indicated durations. (C, D) ELISA analysis of TGF-β1 levels in culture supernatants following LL37 stimulation (C) or with SIS3 or Stattic pretreatment (D). (E) Schematic diagram of a transwell-based macrophage-fibroblast coculture system (created with BioRender.com). (F, G) WB analysis of α-SMA expression (F) and EdU incorporation assay with quantification of EdU⁺ cells (G) in HDFs after coculture with macrophages. (H) Schematic diagram of macrophage depletion using CL in LL37-treated mice. (I, J) Representative skin manifestations at different time points (I), and quantification of skin lesion area ratio over time, compared with LL37+CL group at day 7 (J). (K) Quantification of dermal infiltration of F4/80⁺ cells by IHC. (L) Representative H&E staining, with quantification of dermal infiltrating cells and epidermal thickness. (M) Representative Masson's trichrome (upper) and Sirius red (lower) staining, with quantification of collagen thickness, CVF%, and Sirius red-positive area. (N) Representative IHC staining of vimentin (upper) and α-SMA (lower), with quantification of positive area fractions in the dermis. (O) ELISA analysis of TGF-β1 levels in mouse skin tissues. n ≥ 3 for all experiments. Data are presented as mean ± SD; ^*^P < 0.05, ^**^P < 0.01, ^***^P < 0.001; ns, not significant. Scale bar = 60 μm.

We next established an *in vitro* model to determine whether LL37 directly drives MMT. THP-1 monocytes were differentiated into macrophages upon treatment with PMA and then exposed to prolonged LL37 stimulation, which resulted in a myofibroblast-like phenotype characterized by increased *ACTA2* transcription and elevated α-SMA and type I collagen proteins (Fig. 3A-B). Notably, LL37 stimulation significantly increased TGF-β1 secretion in culture supernatants (Fig. 3C), a key profibrotic cytokine. Macrophages were then pretreated with the Smad3 inhibitor SIS3 prior to LL37 stimulation, which markedly attenuated MMT-associated α-SMA expression and TGF-β1 production (Fig. S3A and Fig. 3D). To investigate whether MMT cells influence the surrounding cellular environment, we employed a transwell-based macrophage-fibroblast coculture system (Fig. 3E). In this system, macrophages undergoing MMT promoted fibroblast activation and proliferation, as indicated by increased α-SMA expression and a higher proportion of EdU⁺ cells, whereas these effects were substantially reduced when macrophages were pretreated with SIS3 (Fig. 3F-G and Fig. S3B). These results suggest that prolonged LL37 exposure drives MMT in macrophages, thereby fostering a pro-fibrotic milieu.

Having established that LL37 promotes MMT *in vitro*, we next evaluated the contribution of macrophages to LL37-induced fibrotic remodeling *in vivo*. We therefore employed CL to selectively deplete macrophages *in vivo* (Fig. 3H). This intervention markedly alleviated LL37-induced cutaneous phenotypes, including attenuation of early erythematous responses and subsequent fibrotic thickening of the dorsal skin (Fig. 3I-J). Histological analysis confirmed efficient clearance of dermal macrophages, as reflected by a marked reduction in F4/80⁺ cells (Fig. S3C and Fig. 3K), and demonstrated that their depletion alleviated LL37-induced dermal inflammatory infiltration and epidermal thickening (Fig. 3L). Fibrotic remodeling was also attenuated, as evidenced by reduced collagen deposition and decreased levels of α-SMA and vimentin in the dermis (Fig. 3M-N). Consistently, levels of TGF-β1 in skin tissues were significantly reduced upon macrophage clearance (Fig. 3O). Collectively, these findings support a model in which macrophages function both as precursors of α-SMA⁺ myofibroblast-like cells and as amplifiers of a TGF-β1-dominated profibrotic niche in LL37-induced skin remodeling, such that their depletion attenuates both the inflammatory and fibrotic manifestations.

### Bruceine A attenuates LL37-induced inflammatory and fibrotic remodeling *in vivo*

**Figure 4:**
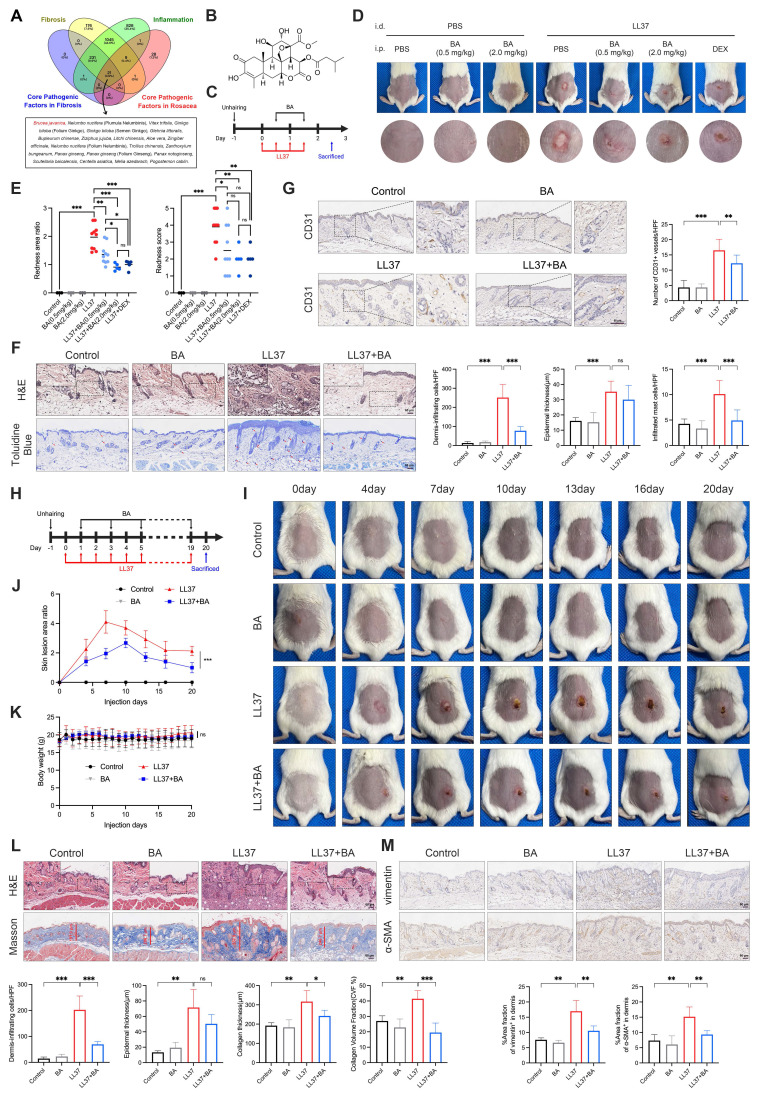
BA attenuates LL37-induced inflammation and fibrosis *in vivo*. (A) Venn diagram of candidate agents identified from ITCM, TCMSP, and HIT databases. (B) Chemical structure of BA. (C) Schematic diagram of BA treatment in the short-term model. (D, E) Representative skin manifestations after treatment with BA at different concentrations and DEX as a positive control (D), with quantification of redness area ratio (left, compared with LL37+DEX) and redness score (right) (E). (F) Representative H&E (upper) and toluidine blue (lower) staining, with quantification of dermal infiltrating cells, epidermal thickness, and infiltrated mast cells. (G) Representative IHC staining of CD31, with quantification of CD31⁺ vessels. (H) Schematic diagram of BA treatment in the long-term model. (I-K) Representative skin manifestations at different time points (I), quantification of skin lesion area ratio over time compared with the LL37+BA group at day 20 (J), and body weight changes (K). (L) Representative H&E (upper) and Masson's trichrome (lower) staining, with quantification of dermal infiltrating cells, epidermal thickness, collagen thickness, and CVF%. (M) Representative IHC staining of vimentin (upper) and α-SMA (lower), with quantification of positive area fractions in the dermis. n ≥ 3 for all experiments. Data are presented as mean ± SD; *P < 0.05, **P < 0.01, ***P < 0.001; ns, not significant. Scale bar = 60 μm.

Given our goal of therapeutically targeting rosacea by concurrently modulating inflammation and fibrosis, we next performed a database-driven screening to identify candidate agents with dual activity, beginning with entries retrieved from ITCM that were annotated to target both fibrosis and inflammation. This set was subsequently refined in TCMSP by retaining those annotated to target all genes within the Core Pathogenic Factors in Fibrosis, while HIT was queried to identify agents annotated to target all genes within the Core Pathogenic Factors in Rosacea. Integration of these datasets yielded 21 overlapping candidates (Fig. 4A). Among these, *Brucea javanica*—and particularly its active constituent BA—stood out, supported by substantial evidence of anti-inflammatory and anti-fibrotic effects (Fig. 4B)^42^-^46^.

We first evaluated the therapeutic efficacy of BA on rosacea-like dermatitis. In the short-term model, intraperitoneal administration of BA markedly alleviated LL37-induced erythema (Fig. 4C-D). Quantitative analyses demonstrated that even low-dose BA (0.5 mg/kg) significantly reduced both the redness area ratio and redness score, with high-dose BA (2.0 mg/kg) achieving efficacy comparable to DEX, a positive control (Fig. 4E). Consistently, histological analyses further demonstrated that low-dose BA alleviated LL37-induced inflammatory alterations: dermal inflammatory-cell infiltration and epidermal thickness were both increased after LL37 challenge, while BA significantly reduced infiltration and tended to decrease epidermal thickness, although not reaching statistical significance (Fig. 4F). LL37-induced mast-cell accumulation was partially attenuated by BA treatment (Fig. 4F), whereas the increase in CD31⁺ vessels—another characteristic feature of rosacea—was also suppressed (Fig. 4G). These findings collectively demonstrate that BA exerts a broad inhibitory effect on LL37-induced inflammatory responses associated with rosacea.

We next assessed whether BA exerted the expected anti-fibrotic effect (Fig. 4H). During long-term LL37 induction, erythema expanded to a maximum at approximately day 7, followed by gradual contraction associated with fibrotic remodeling. Prolonged low-dose BA administration markedly limited early lesion expansion and attenuated subsequent fibrotic phenotype (Fig. 4I-J). No significant alterations in body weight or major organs were observed, indicating good tolerability of BA (Fig. 4K and Fig. S4A). Histological assessment demonstrated that long-term LL37 exposure induced dense dermal infiltration, increased epidermal thickness, and marked collagen accumulation. BA treatment alleviated dermal infiltration and reduced collagen deposition, with both collagen thickness and collagen volume fraction significantly decreased relative to the LL37 group (Fig. 4L). Moreover, chronic LL37 exposure upregulated vimentin and α-SMA in the dermis, indicative of fibroblast activation and myofibroblast differentiation, which were largely suppressed by BA (Fig. 4M). Together, these findings demonstrate that persistent LL37 stimulation drives both inflammatory and fibrotic remodeling of the skin, while BA effectively counteracts these processes.

### Bruceine A attenuates MMT-associated fibrosis in the dermis and mitigates keratinocyte-mediated inflammation in the epidermis

**Figure 5:**
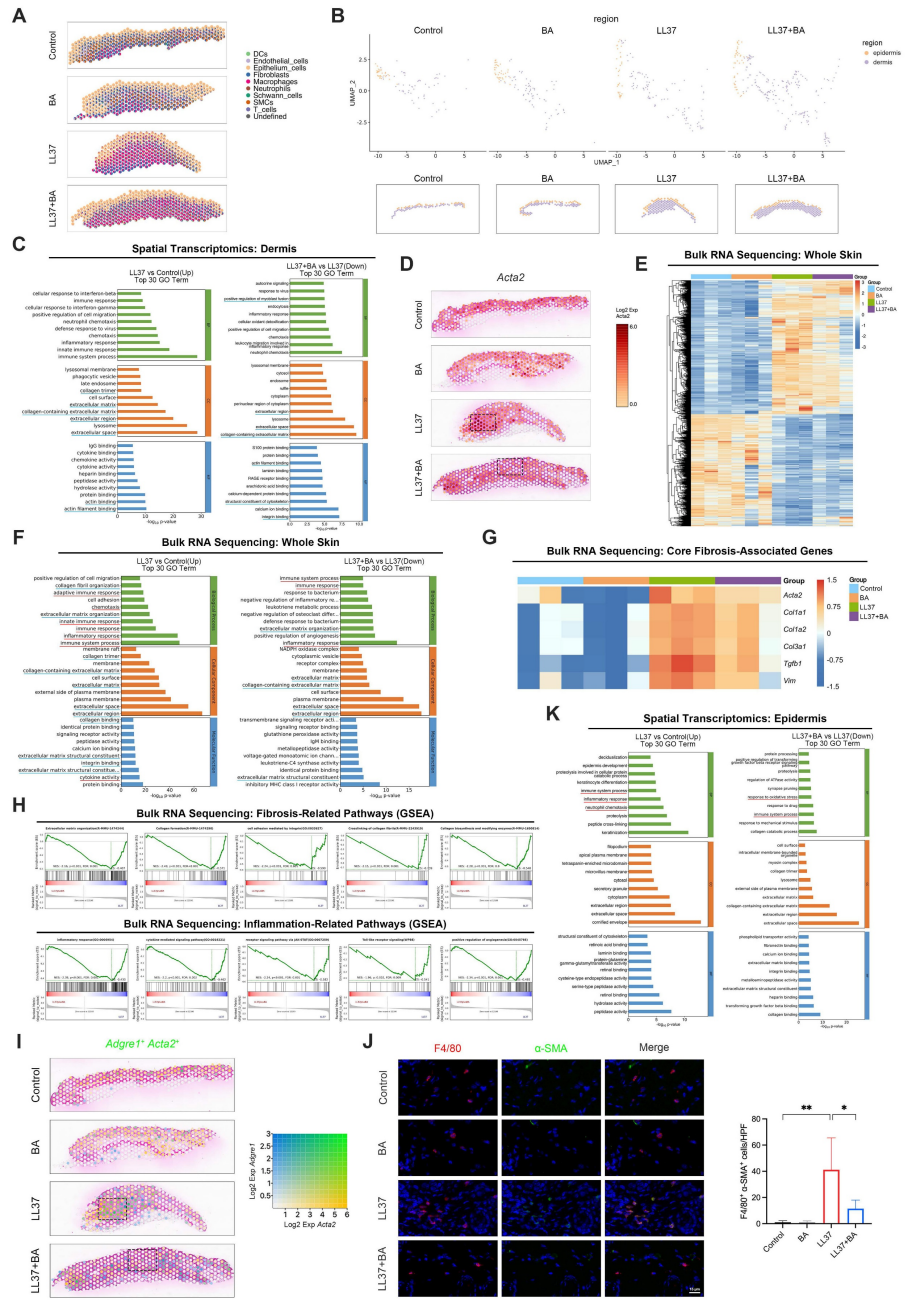
BA attenuates dermal MMT-associated fibrosis and epidermal inflammation**.** (A) RCTD-inferred cell type composition across spatial spots from mouse skin ST. (B) ST maps delineating epidermal and dermal regions. (C) GO enrichment analysis performed on the dermal region from mouse ST, comparing LL37 versus control (left) and LL37+BA versus LL37 (right). (D) Spatial feature plots of *Acta2*. (E-H) Bulk RNA-seq analysis of mouse skin (n = 3). (E) Heatmap of significantly differentially expressed genes across groups. (F) GO enrichment analysis performed on whole skin, comparing LL37 versus control (left) and LL37+BA versus LL37 (right). (G) Heatmap of genetic profiles associated with fibrosis across groups. (H) GSEA showing fibrosis-related pathways (upper) and inflammation-related pathways (lower) in the comparison of LL37+BA versus LL37. (I) Spatial feature plots of co-expression of *Adgre1* and *Acta2*. (J) Representative mIHC images of F4/80 (red), α-SMA (green), and DAPI (blue) in the long-term mouse model with or without BA treatment, with quantification of F4/80⁺ α-SMA⁺ cells (n = 6). (K) GO enrichment analysis performed on the epidermal region from mouse ST, comparing LL37 versus control (left) and LL37+BA versus LL37 (right). Blue underlines (C, F), fibrosis-related terms; red underlines (F, K), inflammation-related terms. Data are presented as mean ± SD; *P < 0.05, **P < 0.01, ***P < 0.001; ns, not significant. Scale bar = 15 μm.

To characterize the spatial landscape of chronic remodeling, we further analyzed the dorsal skin tissues from the long-term LL37-induced model described above using 10x Genomics Visium CytAssist ST across control, BA, LL37, and LL37+BA groups. Unsupervised clustering of the ST profiles identified six transcriptionally distinct domains in the tissue (Fig. S5A-S5B). Considering that each captured spot contains multiple cells, we next integrated the ST and scRNA-seq data using robust cell type decomposition (RCTD) to estimate the proportions of different cell types within each spot. This analysis revealed that under long-term LL37 stimulation, macrophages became the dominant cell type in most spots, an effect that was partially ameliorated by BA (Fig. 5A).

Fibrosis in rosacea predominantly occurs within the dermis, whereas inflammation is largely mediated by keratinocytes. Therefore, we delineated the epidermis and dermis in the ST data and first focused on the dermal region (Fig. 5B). Compared with the control group, the LL37 group showed upregulated genes predominantly associated with fibrotic remodeling, such as collagen trimer, collagen-containing ECM, extracellular region, and related terms. Conversely, downregulated genes in the LL37+BA group relative to LL37 alone were significantly enriched in ECM-related pathways, indicating that BA treatment attenuated LL37-induced fibrotic processes (Fig. 5C). These findings were further validated by* Acta2* spatial mapping, which showed an expansion of dermal regions in the LL37 group that was reduced after BA treatment (Fig. 5D).

To validate these observations, transcriptomic profiling was performed on whole-skin samples (n = 3 per group). The heatmap showed extensive transcriptional alterations induced by LL37, while BA treatment partially alleviated these changes (Fig. 5E). Consistent with the ST findings, functional enrichment showed prominent activation of fibrosis-related pathways, including ECM organization, collagen-containing ECM, and ECM structural constituent, which were reduced in the LL37+BA group (Fig. 5F). Core fibrosis-associated genes, including *Acta2, Col1a1, Col1a2, Col3a1, Tgfb1,* and *Vim*, were strongly induced by LL37 and decreased to varying extents with BA treatment (Fig. 5G). GSEA demonstrated that fibrosis-related pathways, including collagen formation, crosslinking of collagen fibrils, and collagen biosynthesis and modifying enzymes, were significantly suppressed in the LL37+BA group compared with LL37 alone (Fig. 5H, upper panel).

As demonstrated earlier in this study, MMT contributes to fibrotic remodeling in rosacea. We next examined whether BA could interfere with this process *in vivo*. Spatial transcriptomics revealed that LL37-treated skin exhibited increased regions with co-expression of *Adgre1* and *Acta2*, whereas these regions were markedly reduced following BA administration (Fig. 5I). Analysis with mIHC further confirmed that BA suppressed the accumulation of F4/80⁺ α-SMA⁺ cells induced by LL37 (Fig. 5J and Fig. S2D). These results provide essential validation that BA alleviates MMT *in vivo*, thereby targeting a central pathogenic mechanism of fibrotic remodeling.

Within the epidermal region, long-term LL37 elicited prominent inflammatory activation, with enrichment of immune system process, inflammatory response, and neutrophil chemotaxis; these inflammatory programs were partially reduced following BA treatment (Fig. 5K). Consistently, bulk RNA-seq recapitulated the LL37-induced upregulation of immune and chemotactic programs and showed a reduction in the LL37+BA group (Fig. 5F). GSEA further indicated that inflammation-related pathways, including inflammatory response, cytokine-mediated signaling pathway, and receptor signaling pathway via JAK-STAT, were negatively enriched in LL37+BA versus LL37 (Fig. 5H, lower panel).

Together, these data demonstrate that long-term LL37 stimulation induces MMT and subsequent fibrotic remodeling in the dermis, while simultaneously activating inflammatory programs in the epidermis, thereby recapitulating features of human rosacea (Fig. S5C). Importantly, BA treatment attenuates MMT-associated fibrosis and keratinocyte-mediated inflammation, supporting its dual role in suppressing inflammation and fibrosis.

### Bruceine A targets STAT3 as a pivotal regulator in rosacea pathogenesis

**Figure 6:**
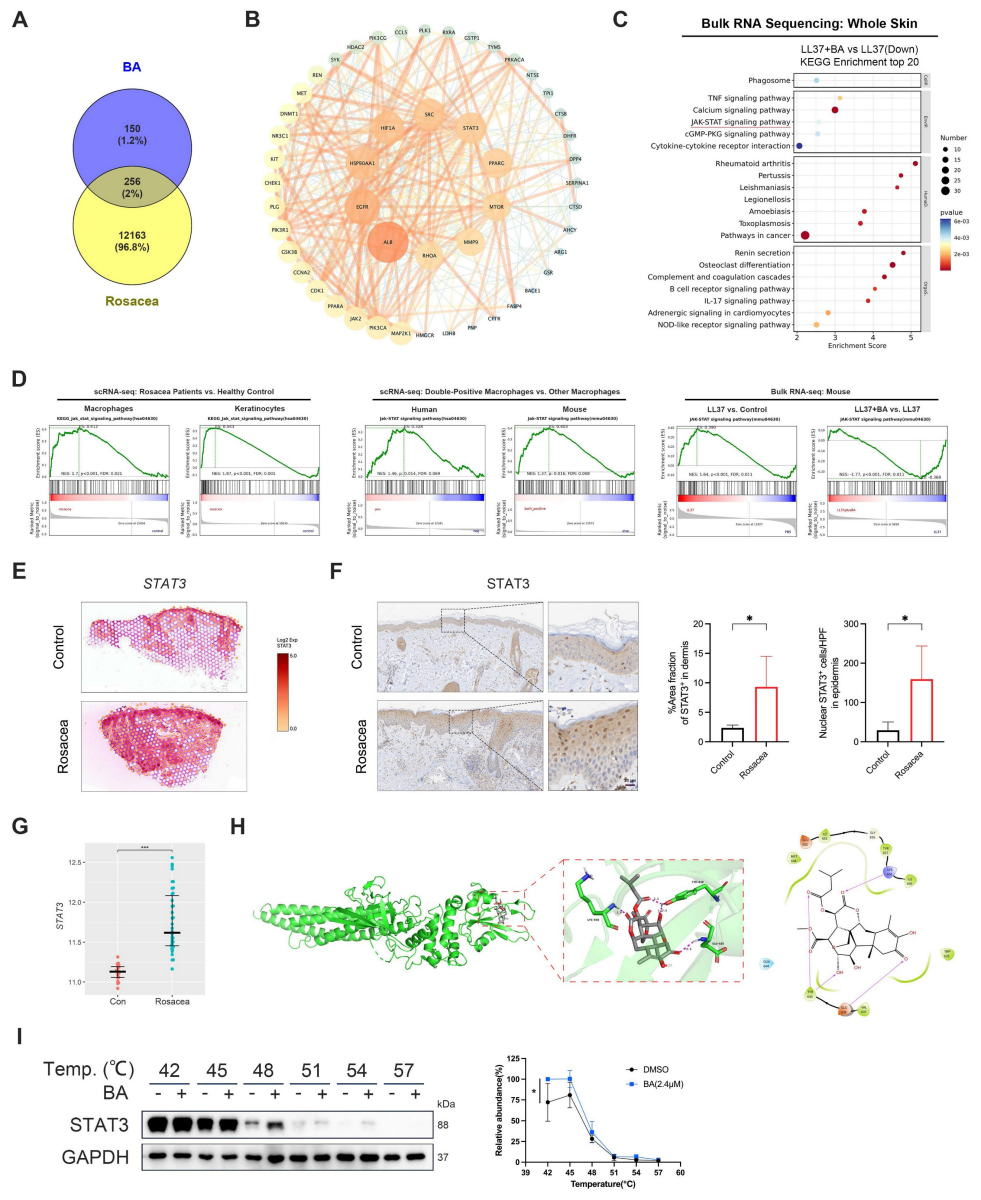
BA directly targets STAT3 in rosacea. (A) Venn diagram showing the common targets of BA and rosacea. (B) PPI network of potential targets for BA therapy of rosacea. (C) KEGG enrichment analysis of LL37+BA versus LL37 from bulk RNA-seq analysis of mouse skin. The JAK-STAT signaling pathway is indicated by a red underline. (D) GSEA showing enrichment of the JAK-STAT signaling pathway across datasets, including scRNA-seq of macrophages and keratinocytes from rosacea patients versus healthy controls (left), scRNA-seq comparing double-positive macrophages with other macrophages in human and mouse (middle), and bulk RNA-seq of mouse skin comparing LL37 versus control and LL37+BA versus LL37 (right). (E) Spatial feature plots of *STAT3* in human skin. (F) Representative IHC staining of STAT3 in rosacea patients and healthy controls, with quantification of dermal STAT3⁺ area fraction and epidermal nuclear STAT3⁺ cells (n ≥ 4). (G) Relative expression levels of *STAT3* from GEO dataset GSE65914. (H) The 3D representation of BA binding to STAT3 protein (docking score: -4.388), along with the planar amino acid interactions. (I) CETSA of STAT3 with BA treatment, showing thermal stability detected by Western blot, with quantification of normalized STAT3 levels (n = 3). Data are presented as mean ± SD; *P < 0.05, **P < 0.01, ***P < 0.001; ns, not significant. Scale bar = 20 μm.

Building on these observations, we investigated the molecular mechanisms underlying BA's effects by predicting putative targets using PharmMapper and SuperPred. Integrating these predictions with rosacea-associated genes from the GEO dataset GSE65914 yielded 256 overlapping targets (Fig. 6A). These targets were subsequently subjected to PPI analysis in the STRING database. The resulting network revealed several hub nodes, including STAT3, SRC, EGFR, MTOR and MMP9, which may represent critical mediators of BA's therapeutic activity in rosacea (Fig. 6B). Bulk RNA-seq further showed that BA-downregulated genes were significantly enriched in inflammation-related pathways, with the JAK-STAT signaling pathway identified as one of the top enriched pathways (Fig. 6C), converging with the STAT3 hub revealed in the PPI network.

Consistent with this prediction, analysis of human scRNA-seq data demonstrated significant enrichment of the JAK-STAT signaling pathway in both macrophages and keratinocytes from rosacea lesions compared with healthy controls (Fig. 6D, left panel). Moreover, *CD68*⁺ *ACTA2*⁺ macrophages in human skin exhibited stronger JAK-STAT activity than other macrophages, a pattern that was also observed in *Adgre1*⁺ *Acta2*⁺ macrophages in mice (Fig. 6D, middle panel). Bulk RNA-seq of mouse skin further showed robust induction of this pathway after LL37 stimulation, whereas BA administration effectively suppressed its activation (Fig. 6D, right panel). ST analysis of human skin further revealed increased STAT3 expression in the dermis of rosacea lesions compared with healthy skin (Fig. 6E). IHC demonstrated expanded STAT3⁺ regions in the dermis and greater nuclear accumulation of STAT3 in epidermal keratinocytes in rosacea (Fig. 6F). Analysis of external datasets further confirmed elevated STAT3 transcript levels in rosacea relative to healthy controls, particularly in PhR (Fig. 6G and Fig. S6A). Molecular docking analysis of JAK1, JAK2, TYK2, and STAT3 identified STAT3 as the most likely binding partner of BA (Fig. 6H and Fig. S6B-S6D). Consistently, CETSA showed that BA stabilized STAT3, supporting a direct interaction (Fig. 6I). Collectively, these findings indicate that aberrant JAK-STAT activation in both macrophages and keratinocytes constitutes a hallmark of rosacea pathogenesis, and identify STAT3 not only as a central signaling hub but also as a direct molecular target of BA *in vivo*.

### Bruceine A suppresses STAT3 palmitoylation to inhibit MMT and inflammation

**Figure 7:**
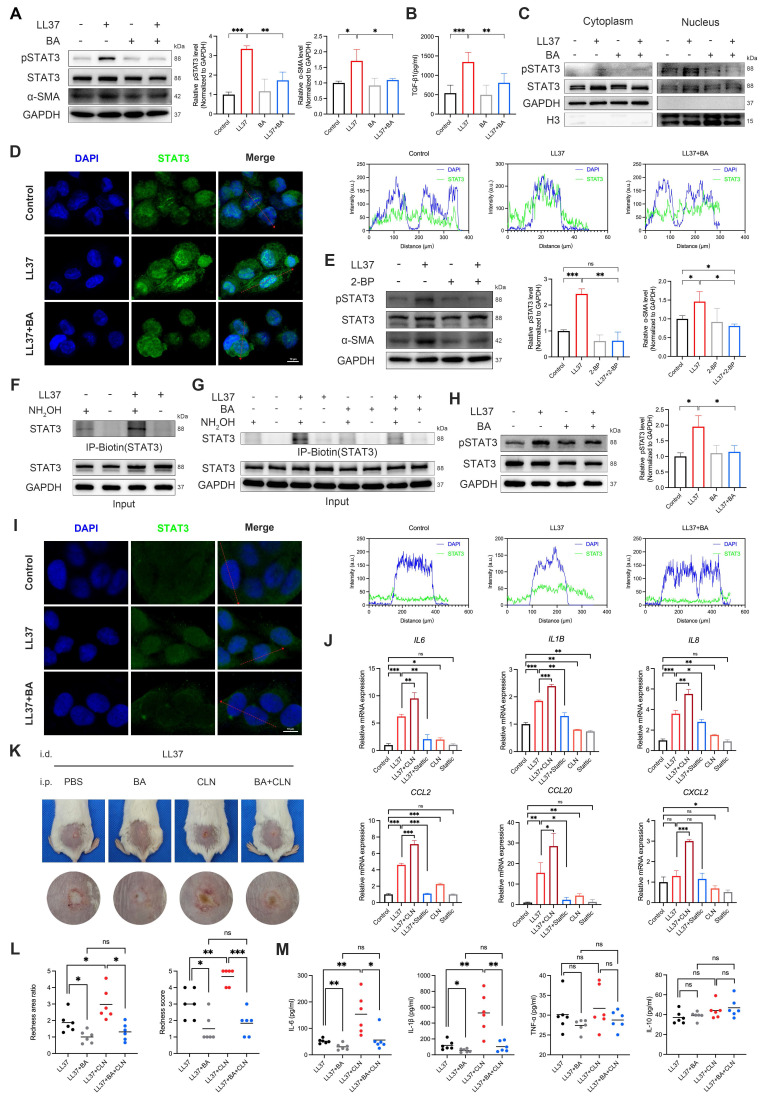
BA interferes with STAT3 palmitoylation and activation, suppressing MMT and inflammatory responses. (A-D) THP-1-derived macrophages were stimulated with LL37, with or without BA pretreatment. (A) WB analysis of p-STAT3 (Tyr705), STAT3, and α-SMA. (B) ELISA analysis of TGF-β1 levels in culture supernatants. (C) WB analysis of cytoplasmic and nuclear fractions showing the distribution of STAT3 and p-STAT3. (D) Representative confocal microscopy images showing the distribution of STAT3 (green) and DAPI (blue), along with line-scan analysis assessing their colocalization. (E) WB analysis of p-STAT3 (Tyr705), STAT3, and α-SMA in macrophages after LL37 stimulation, with or without 2-BP pretreatment. (F, G) ABE assays showing the levels of palmitoylated STAT3 in macrophages after LL37 stimulation (F) or with BA pretreatment (G). (H, I) HaCaT cells were stimulated with LL37, with or without BA pretreatment. (H) WB analysis of p-STAT3 (Tyr705) and STAT3. (I) Representative confocal microscopy images showing the distribution of STAT3 (green) and DAPI (blue), along with line-scan analysis assessing their colocalization. (J) Relative mRNA expression of *IL6*, *IL1B*, *IL8*, *CCL2*, *CCL20*, and *CXCL2* in HaCaT cells after LL37 stimulation, with or without treatment with CLN or Stattic. (K, L) Representative skin manifestations in the LL37-induced rosacea-like dermatitis model with PBS, BA, CLN, or BA+CLN (K), and quantification of redness area ratio (left, compared with LL37+BA) and redness score (right) (L). (M) Multiplex secretome analysis of tissue homogenate supernatants showing the levels of IL-6, IL-1β, TNF-α, and IL-10 across groups. n ≥ 3 for all experiments. Data are presented as mean ± SD; ^*^P < 0.05, ^**^P < 0.01, ^***^P < 0.001; ns, not significant. Scale bar = 10 μm.

Given that the JAK-STAT pathway is enriched in rosacea lesions and in double-positive macrophages (Fig. 6D), we hypothesized that aberrant STAT3 signaling contributes to MMT, and that BA interferes with this process by attenuating STAT3 activation. LL37 markedly enhanced STAT3 phosphorylation at Tyr705 together with increased α-SMA expression in macrophages, both of which were significantly reduced by BA (Fig. 7A). This was accompanied by robust induction of TGF-β1 secretion, which was likewise attenuated by BA (Fig. 7B). Consistently, pretreatment with Stattic, a STAT3 inhibitor, similarly attenuated LL37-induced MMT-associated responses, including reduced α-SMA expression, TGF-β1 production, and the ability of macrophages to promote fibroblast activation and proliferation (Fig. 3D-G and Fig. S3A-S3B). Moreover, we demonstrated that LL37 promotes STAT3 nuclear translocation, whereas BA effectively restricted this process (Fig. 7C-D).

Previous evidence indicates that palmitoylation controls STAT3 membrane anchoring and nuclear translocation^35^, ^36^; therefore, we next assessed whether this modification underlies LL37-induced STAT3 phosphorylation. Treatment with 2-BP, a palmitoylation inhibitor, reduced LL37-induced STAT3 phosphorylation and α-SMA expression in macrophages (Fig. 7E). To directly assess STAT3 palmitoylation, we performed ABE assays, which showed that LL37 stimulation enhanced palmitoylation of STAT3 (Fig. 7F). However, BA treatment reduced LL37-induced STAT3 palmitoylation (Fig. 7G). In summary, these results establish that STAT3 palmitoylation is a prerequisite for LL37-induced STAT3 activation and subsequent MMT, and demonstrate that BA effectively inhibits this process.

Having established its contribution to MMT, we next assessed the role of STAT3 in inflammatory activation. LL37 rapidly induced STAT3 phosphorylation in keratinocytes (Fig. S7A), which was suppressed by BA (Fig. 7H). Confocal microscopy further demonstrated that LL37 promoted nuclear translocation of STAT3, whereas BA restricted this process (Fig. 7I). As expected, LL37 also markedly upregulated the transcription of multiple inflammatory mediators, including *IL6*, *IL1B*, *IL8*, *CCL2*, and *CCL20*; this response was attenuated by Stattic, whereas CLN, a STAT3 activator, further enhanced these responses (Fig. 7J). Notably, in the rosacea-like dermatitis model, BA effectively counteracted CLN-enhanced inflammatory responses, as evidenced by improvement of erythema and reduced expression of proinflammatory mediators, including IL-6 and IL-1β (Fig. 7K-M).

Together, these findings establish STAT3 palmitoylation as a prerequisite for its activation, and highlight STAT3 as a central driver of both MMT and keratinocyte-mediated inflammation in rosacea, with BA effectively counteracting these pathogenic processes by directly targeting STAT3 and preventing its palmitoylation.

## Discussion

A defining feature of cutaneous fibrosis is the persistence of activated myofibroblasts, which orchestrate excessive ECM production and remodeling, ultimately leading to distortion of the normal tissue architecture and loss of function^47^. Within this framework, PhR is regarded as a fibrosis-associated subtype of rosacea, characterized by dermal thickening and tissue remodeling, with sebaceous gland hyperplasia as a frequent concomitant finding^48^, ^49^. Extending this concept, we observed a tendency toward fibrotic remodeling already in non-phymatous subtypes of rosacea, where inflammation predominates, supported by serum proteomics revealing that TGF-β1 exhibited one of the most pronounced increases and effectively discriminated patients from healthy controls (AUC = 0.93) (Fig. 1A); validation in external GEO datasets and ST analysis of patient skin showing increased expression of fibrosis-associated markers; and histological evidence of enhanced α-SMA staining within the dermis. Therapeutic options for PhR, in which fibrosis is already established, remain inadequately supported by robust evidence. Conventional interventions, including ablative laser therapies or surgical approaches, can achieve cosmetic improvement but may require multiple sessions and carry risks of scarring and hypopigmentation^50^, ^51^. These limitations highlight the importance of initiating anti-inflammatory and anti-fibrotic strategies at early stages, before chronic inflammation progresses to irreversible fibrosis.

Having established that dermal fibrosis is integral to rosacea, the next question is the cellular origin of the myofibroblasts that drive this remodeling. While most myofibroblasts arise from resident fibroblasts, additional sources have been recognized, including the newly identified MMT^17^-^20^. In line with this possibility, rosacea lesions exhibit pronounced dermal macrophage infiltration^7^, ^9^, ^29^, and our analyses—drawing on external transcriptomic datasets, spatial profiling, scRNA-seq, and mIHC—consistently demonstrated macrophage expansion in lesional skin, as shown in Fig. 1 and Fig. S1. Together, these observations suggest that MMT may participate in the fibrotic remodeling of rosacea.

A characteristic feature of MMT cells is the simultaneous co-expression of macrophage markers such as CD68 or F4/80 and the myofibroblast-associated protein α-SMA, and these cells frequently exhibit an M2 phenotype together with the ability to produce ECM components such as collagen I^21^, ^22^, ^52^. In human skin, scRNA-seq identified a subset of macrophages undergoing transition toward a myofibroblast-like state, and these *CD68*⁺ *ACTA2*⁺ macrophages displayed a more fibrogenic tendency (Fig. 1F-O). On UMAP embeddings, *CD68*⁺ *ACTA2*⁺ cells appeared mainly within the myeloid and fibroblast subclusters, a distribution consistent with a gradual, continuum-like shift in identity traced by pseudotime from macrophage-like toward fibroblast-like states. Histologic assessment provided *in situ* evidence of CD68⁺ α-SMA⁺ cells, supporting the occurrence of MMT. Furthermore, pathological evidence indicated a progressive increase along the clinical spectrum from inflammation-predominant subtypes (ETR, PPR) to fibrosis-predominant phymatous disease (PhR).

Aberrant accumulation of LL37 is a well-recognized pathogenic driver in rosacea, provoking keratinocyte activation, immune cell recruitment, and inflammation^7^, ^8^. Parallel observations in the LL37-induced mouse models, consistent with our findings in humans and as shown in Fig. 2, further support the relevance of MMT to rosacea. Macrophages co-expressing α-SMA (F4/80⁺ α-SMA⁺, consistent with *Adgre1*⁺ *Acta2*⁺ transcriptomic signatures) emerged at early stages of LL37 exposure and expanded progressively with chronic stimulation, indicating that sustained inflammation not only initiates but also reinforces their fibrogenic reprogramming. Importantly, lineage tracing with *Cx3cr1*-GFP knock-in mice provided direct *in vivo* evidence that myofibroblast-like cells can indeed arise from macrophages. Rather than representing a transient marker overlap, the stepwise accumulation of *Cx3cr1*-GFP⁺ α-SMA⁺ cells paralleled dermal collagen thickening, underscoring a causal link between macrophage plasticity and cutaneous fibrotic remodeling. *In vitro* experiments further indicated that LL37 could reprogram macrophages toward a myofibroblast-like state and was associated with increased TGF-β1 release. This observation is in line with reports identifying TGF-β signaling as a central driver of fibrosis in PhR^53^-^55^, with TGF-β1 also recognized as a key mediator of MMT^15^, ^19^, ^22^, ^37^. In this context, functional inhibition and macrophage-fibroblast coculture analyses revealed that activation of MMT programs in macrophages is associated with a profibrotic phenotypic shift and contributes to their ability to promote fibroblast activation and proliferation. Thus, the fibrogenic role of MMT cells involves not only their direct contribution to ECM synthesis and secretion^56^, but also the maintenance of a TGF-β1-dominated niche that can amplify fibrotic remodeling. Consistently, macrophage depletion alleviated inflammation, lowered tissue TGF-β1 levels and reduced collagen deposition (Fig. 3), supporting MMT as a link between inflammation and the amplification of fibrosis.

Despite the expanding therapeutic armamentarium for rosacea, current options remain suboptimal. Conventional anti-inflammatory agents such as tetracyclines alleviate papules and pustules but have limited effects on persistent erythema and rarely modify the underlying disease course^57^. Isotretinoin can improve granulomatous and early soft phymatous changes, yet teratogenicity necessitates caution in prolonged use^58^-^61^. Physical interventions, including ablative laser treatments and surgical excision for phymatous changes, as well as vascular lasers or intense pulsed light for erythema and telangiectasia, can provide cosmetic improvement; however, they usually require multiple sessions and may be associated with persistent erythema and swelling, pigmentary alterations, or scarring^58^, ^59^, ^61^. Targeted strategies against vascular or neuroimmune pathways are emerging, but they generally address only certain clinical features of rosacea and seldom achieve durable disease control, often necessitating ongoing or combined treatment^2^, ^58^, ^59^, ^61^, ^62^. These therapeutic shortcomings underscore the urgent need for mechanism-based interventions that can both mitigate inflammation and prevent the fibrotic remodeling underlying advanced disease. Natural products represent a valuable reservoir for such candidates, as many exhibit pleiotropic bioactivities relevant to complex skin disorders^63^-^65^. To this end, we conducted an integrative screening across multiple pharmacological datasets to prioritize compounds with dual anti-inflammatory and anti-fibrotic potential. Guided by this strategy, we focused on BA, a natural quassinoid isolated from *Brucea javanica* with a long history of medicinal use. BA has attracted increasing attention owing to its demonstrated activities in preclinical models of fibrosis and inflammation^42^-^46^, positioning it as a promising candidate for intercepting the dual pathogenic axes of rosacea.

Building on this rationale, our investigation demonstrates that BA provides integrated benefits across the key pathological axes of rosacea. In acute LL37-induced inflammation, BA alleviated erythema, reduced immune cell infiltration — including mast cells, a known contributor to fibroblast activation and fibrosis^66^-^68^ — and limited vascular responses. In the chronic model, BA further prevented dermal collagen accumulation and α-SMA upregulation. These improvements coincided with marked suppression of MMT, underscoring BA's ability to curtail a critical cellular source of myofibroblasts and supporting the notion that BA can restrain the progression from chronic inflammation to irreversible fibrosis (Fig. 4 and Fig. 5). Consistent with previous reports, BA administration did not cause death, significant body weight loss, or other noticeable adverse effects on major organs *in vivo*^42^, ^69^-^72^. At the mechanistic level, we identified STAT3 as a central hub with critical roles in both immune activation and fibrotic remodeling, and demonstrated that BA interferes with this pathway by blocking STAT3 palmitoylation — an essential prerequisite for its phosphorylation and nuclear translocation^35^, ^36^, which is aberrantly enhanced by LL37 in rosacea. By interrupting this modification, BA not only curtailed the ability of macrophages to transdifferentiate into α-SMA⁺ myofibroblasts, but also dampened keratinocyte-driven inflammatory cascades, thereby interrupting the pathological link between immune activation and fibrotic remodeling (Fig. 6 and Fig. 7). These results establish STAT3 palmitoylation as a previously underappreciated pathogenic determinant of rosacea and highlight BA as a prototype therapy that exploits this vulnerability to deliver coordinated anti-inflammatory and anti-fibrotic benefits.

Despite these advances, several limitations should be acknowledged. Although the LL37-induced models are widely used and provide valuable mechanistic insights, they cannot fully recapitulate the chronic and multifactorial nature of human rosacea. Our data support a direct interaction between BA and STAT3; however, as a small-molecule compound, BA may also exert its effects through additional targets beyond STAT3, contributing to the observed phenotypes. In addition, while we demonstrated the therapeutic potential of BA in preclinical models, its translational significance remains to be determined and will require validation in future clinical studies. Altogether, these limitations highlight the need for refined models to fully elucidate the role of MMT in rosacea and to further assess the potential of BA for clinical application.

## Conclusions

In conclusion, this study demonstrates that rosacea has a propensity for dermal fibrosis, already evident when inflammation predominates. We identify MMT as an important contributor that links macrophage infiltration to fibrotic remodeling and progressively expands along the disease-severity trajectory, becoming particularly pronounced toward phymatous change. This process is driven by LL37-induced STAT3 palmitoylation, enabling its phosphorylation and nuclear translocation. Notably, BA inhibited STAT3 palmitoylation, thereby reducing MMT-associated fibrosis and keratinocyte-mediated inflammation. These findings highlight MMT and STAT3 palmitoylation as therapeutic targets and identify BA as a candidate for mechanism-based intervention in rosacea.

## Supplementary Material

Supplementary figures and tables.

## Abbreviations

MMT: macrophage-to-myofibroblast transition
scRNA-seq: single-cell RNA sequencing
ST: spatial transcriptomics
BA: Bruceine A
STAT3: signal transducer and activator of transcription 3
TGF-β1: transforming growth factor beta 1
ETR: erythematotelangiectatic rosacea
PPR: papulopustular rosacea
PhR: phymatous rosacea
OR: ocular rosacea
ECM: extracellular matrix
EMT: epithelial-mesenchymal transition
EndoMT: endothelial-mesenchymal transition
α-SMA: α-smooth muscle actin
JAK: Janus kinase
mIHC: multiplexed immunohistochemistry
SPF: specific pathogen-free
DEX: dexamethasone
CLN: colivelin
CL: clodronate-containing liposomes
PMA: phorbol 12-myristate 13-acetate
2-BP: 2-bromopalmitate
HDF: human dermal fibroblasts
DMEM: Dulbecco's Modified Eagle Medium
BSA: bovine serum albumin
PCA: principal component analysis
UMAP: Uniform Manifold Approximation and Projection
DEGs: differentially expressed genes
FFPE: formalin-fixed paraffin-embedded
PFA: paraformaldehyde
H&E: hematoxylin and eosin
RNA-seq: RNA sequencing
GO: Gene Ontology
KEGG: Kyoto Encyclopedia of Genes and Genomes
GSEA: Gene Set Enrichment Analysis
DAB: 3,3'-diaminobenzidine
TSA: tyramide signal amplification
DAPI: 4',6-diamidino-2-phenylindole
IF: immunofluorescence
GFP: green fluorescent protein
EdU: 5-ethynyl-2'-deoxyuridine
ELISA: Enzyme-Linked Immunosorbent Assay
WB: Western blotting
qPCR: Quantitative real-time polymerase chain reaction
cDNA: complementary DNA
PPI: protein-protein interaction
CETSA: Cellular Thermal Shift Assay
ABE: Acyl-Biotin Exchange
IP: immunoprecipitation
SD: standard deviation
ROC: receiver operating characteristic
AUC: area under the curve
SMCs: smooth muscle cells
DCs: dendritic cells
RCTD: Robust Cell Type Decomposition
