# Supplementary material for: Targeting Macrophage-to-Myofibroblast Transition Mitigates Progression from Inflammation to Fibrosis in Rosacea
Source: Int J Biol Sci. 2026 Feb 4;22(5):2361–82. doi: 10.7150/ijbs.128841 (PMC12965144; doi:10.7150/ijbs.128841)
Supplement: Supplementary file 1 — Supplementary figures and tables. [file ijbsv22p2361s1.pdf]

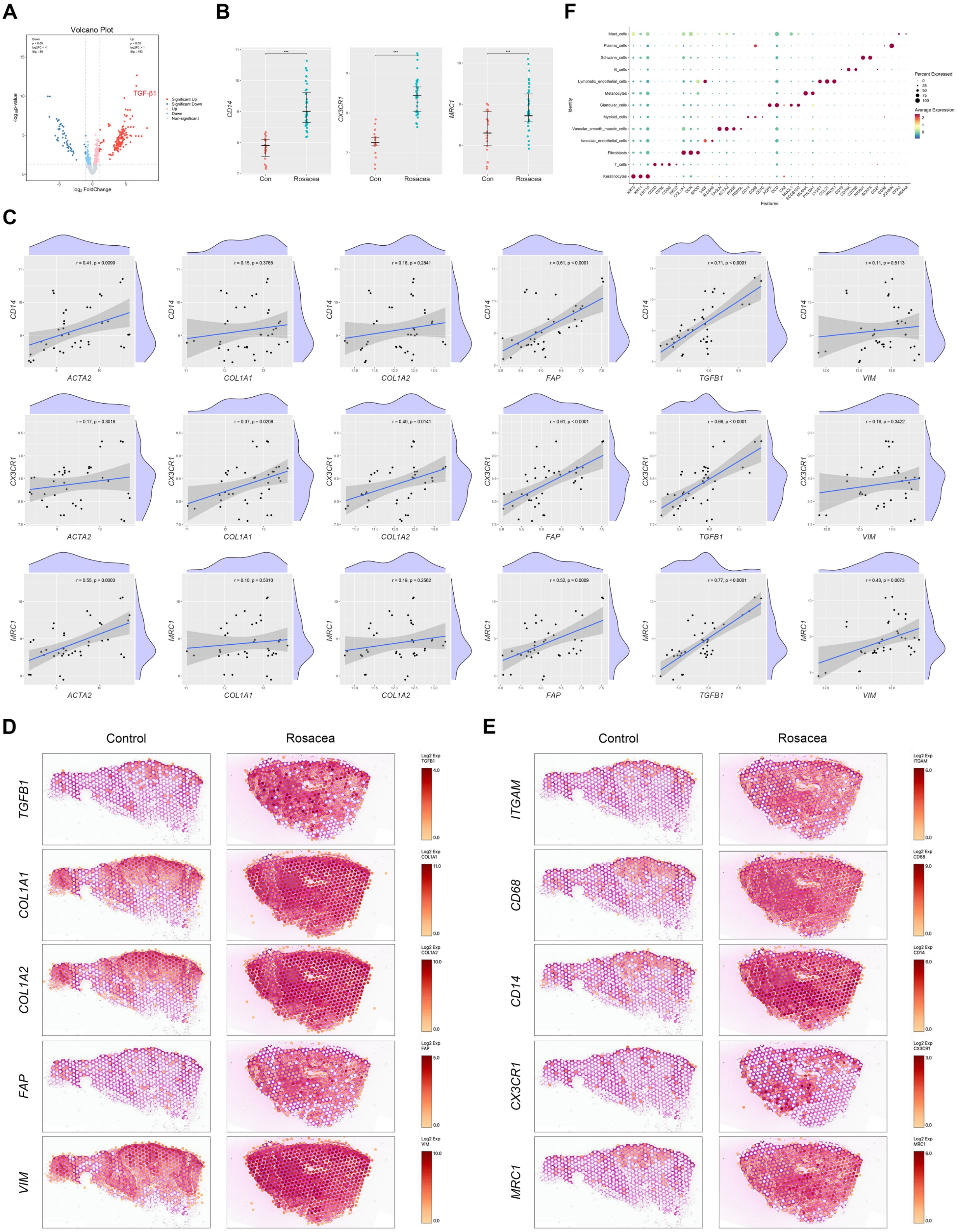
A
Volcano Plot
TGF-β1
Significant Up
Significant Down
Up
Down
Non-significant
log2 FoldChange
B
CD14
CX3CR1
MRC1
Con
Rosacea
F
Mast_cells
Plasma_cells
Schwann_cells
B_cells
Lymphatic_endothelial_cells
Melanocytes
Glandular_cells
Myeloid_cells
Vascular_smooth_muscle_cells
Vascular_endothelial_cells
Fibroblasts
T_cells
Keratinocytes
Identity
Features
Percent Expressed
Average Expression
C
r = 0.41, p = 0.0099
r = 0.15, p = 0.3785
r = 0.18, p = 0.2841
r = 0.61, p < 0.0001
r = 0.71, p < 0.0001
r = 0.11, p = 0.5113
r = 0.17, p = 0.3018
r = 0.37, p = 0.0208
r = 0.40, p = 0.0141
r = 0.61, p < 0.0001
r = 0.66, p < 0.0001
r = 0.16, p = 0.3422
r = 0.55, p = 0.0003
r = 0.10, p = 0.5310
r = 0.19, p = 0.2562
r = 0.52, p = 0.0009
r = 0.77, p < 0.0001
r = 0.43, p = 0.0073
ACTA2
COL1A1
COL1A2
FAP
TGFB1
VIM
D
E
Control
Rosacea
TGFB1
COL1A1
COL1A2
FAP
VIM
ITGAM
CD68
CD14
CX3CR1
MRC1
Log2 Exp

1

Figure S1 Fibrosis and macrophage infiltration are prominent features of rosacea, related to Figure 1. (A) Volcano plot of serum proteomics comparing rosacea patients (n = 18) and healthy controls (n = 19). (B, C) Relative expression levels (B) and correlation analysis (C) from GEO dataset GSE65914. (D, E) Spatial feature plots of fibrosis-associated genes (D) and macrophage-associated genes (E). (F) Dot plot showing representative marker genes for each cell type. Data are presented as mean ± SD; *P < 0.05, **P < 0.01, ***P < 0.001; ns, not significant.

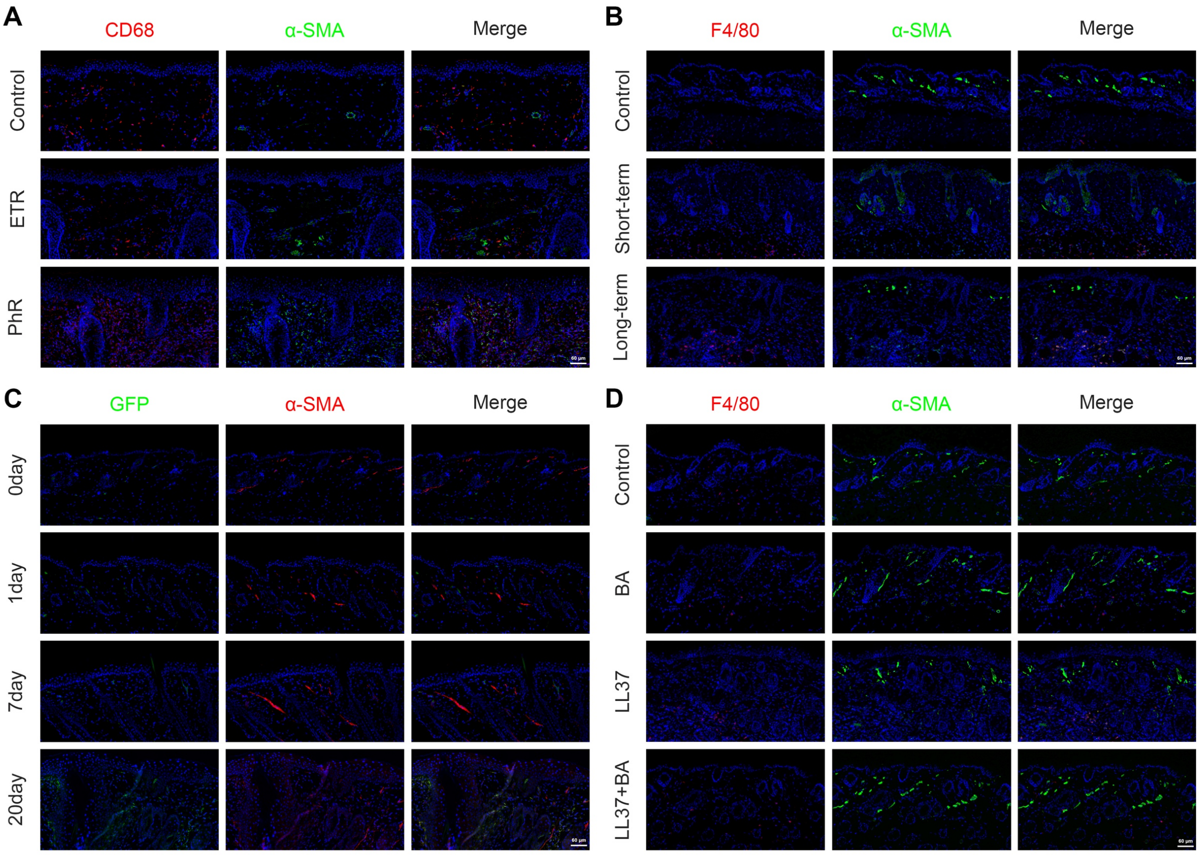

Figure S2 Representative low-magnification mIHC or IF images of MMT under different conditions, related to Figures 1, 2 and 5. (A) CD68 (red), α-SMA (green) and DAPI (blue) in control, ETR and PhR skin. (B) F4/80 (red), α-SMA (green) and DAPI (blue) in control, short-term and long-term mouse models. (C) GFP (green), α-SMA (red) and DAPI (blue) in *Cx3cr1*-GFP knock-in mice subjected to LL37 stimulation for different durations. (D) F4/80 (red), α-SMA (green) and DAPI (blue) in control, BA, LL37 and LL37+BA groups (n ≥ 3). Scale bar = 60 μm.

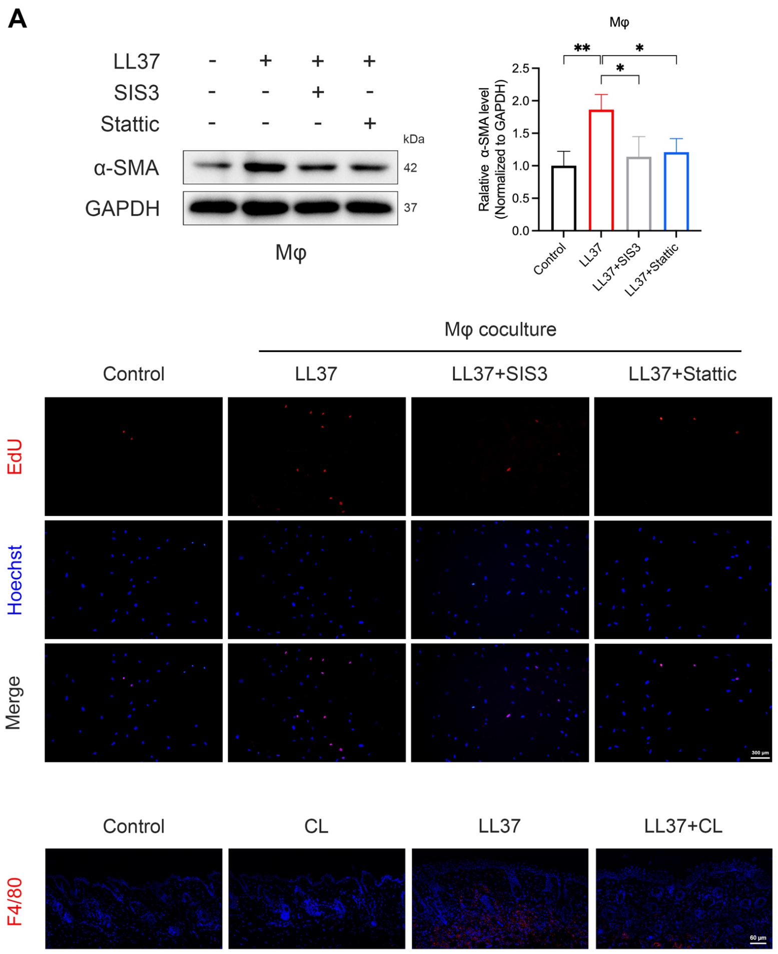

Figure S3 WB analysis and representative EdU and IHC images, related to Figure 3. (A) WB analysis of α-SMA expression in THP-1–derived macrophages following LL37 stimulation with SIS3 or Stattic pretreatment. (B) Representative EdU incorporation assay images in HDFs after coculture with macrophages (EdU, red; Hoechst, blue). (C) Representative IHC images of dermal infiltration of F4/80^{+} cells. $n \geq 3$ for all experiments. Data are presented as mean ± SD; *$P < 0.05$, **$P < 0.01$, ***$P < 0.001$; ns, not significant. Scale bars: 300 μm (B), 60 μm (C).

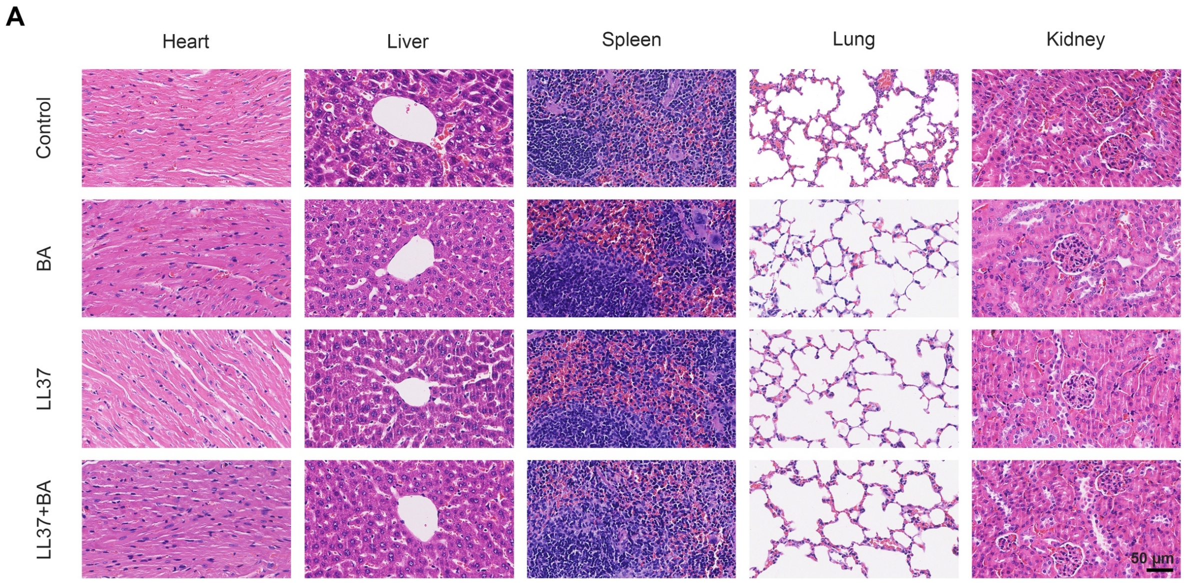

Figure S4 BA shows no systemic adverse effects, related to Figure 4. (A) Representative H&E staining of major organs, including the heart, liver, spleen, lung, and kidney, from the long-term model. Scale bar = 50 μm.

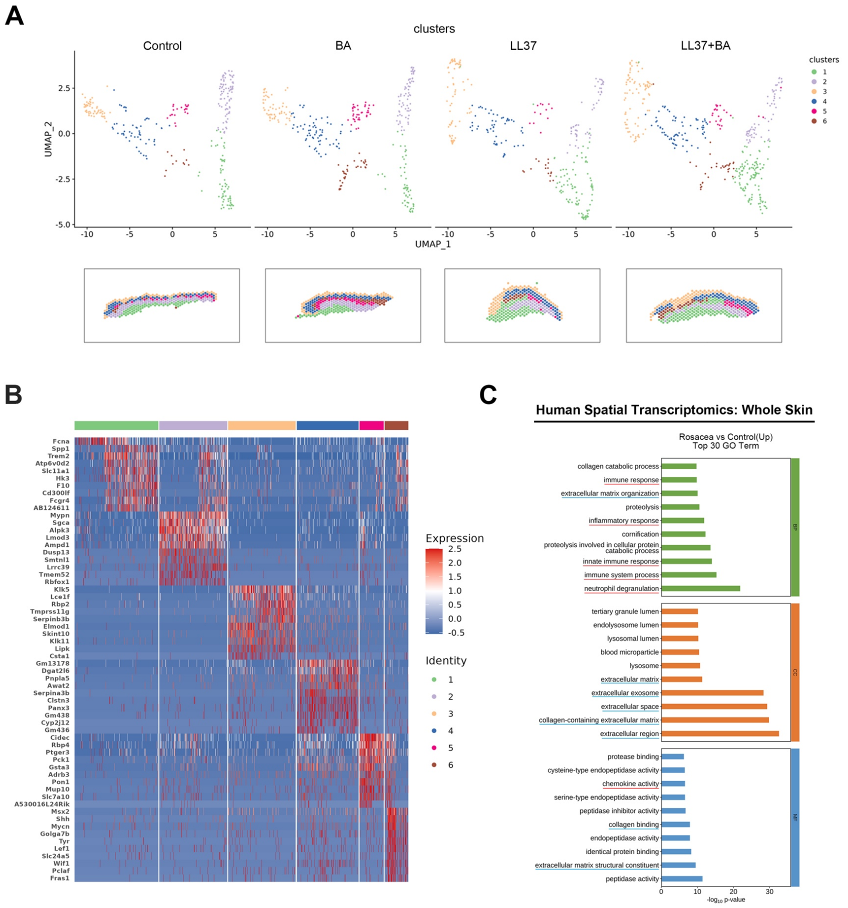

Figure S5 ST analysis of the long-term LL37-induced mouse model with BA treatment and human rosacea, related to Figure 5. (A) UMAP classifying six clusters based on overall gene expression profiles from mouse skin ST. (B) Heatmap showing highly expressed genes in each identified cluster. (C) GO enrichment analysis performed on whole skin from human ST, comparing rosacea and control. Blue underlines, fibrosis-related terms; red underlines, inflammation-related terms.

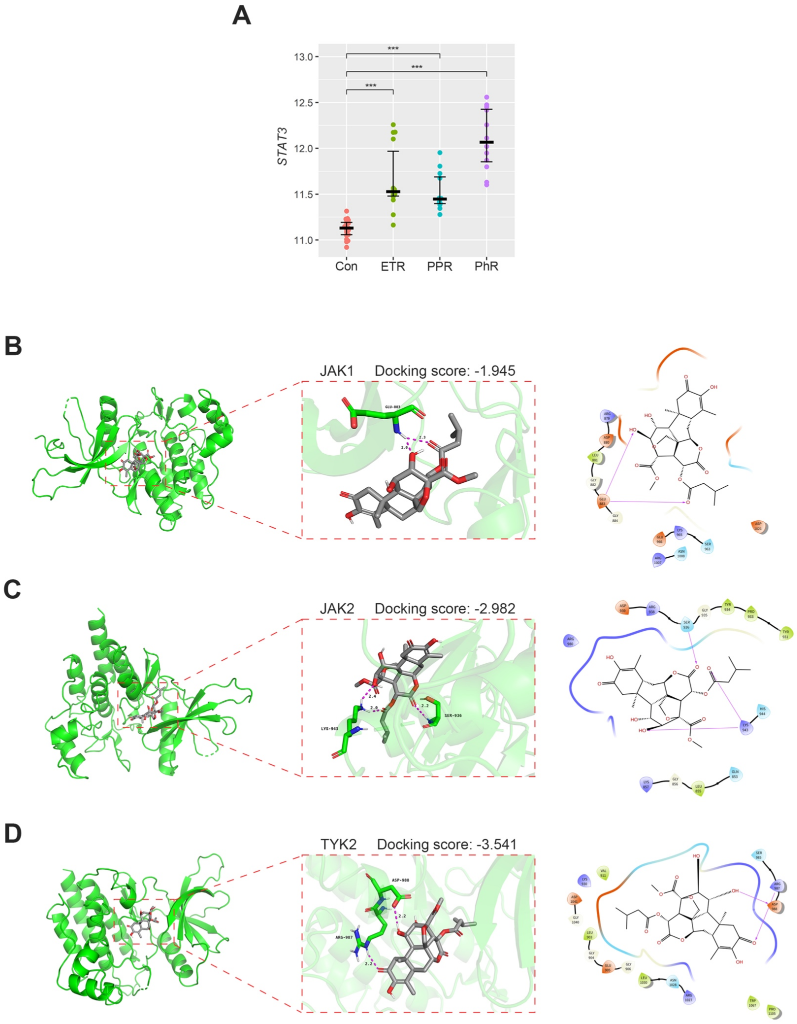

Figure S6 Supplementary analyses of STAT3 expression and BA binding activity, related to Figure 6. (A) Relative expression levels of STAT3 from GEO dataset GSE65914 in rosacea subtypes (ETR, PPR, and PhR) and healthy controls. (B–D) The 3D representations of BA binding to JAK1 (B), JAK2 (C), and TYK2 (D) proteins, along with the planar amino acid interactions. Data are presented as mean ± SD; *P < 0.05, **P < 0.01, ***P < 0.001; ns, not significant.

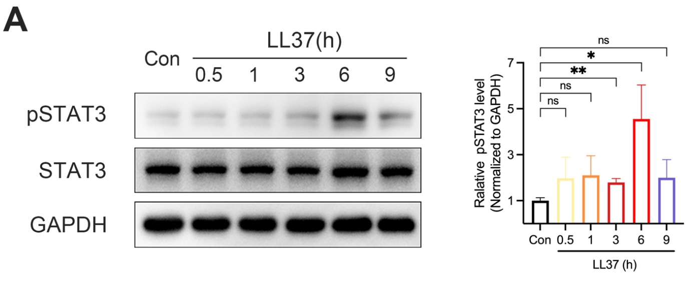

Figure S7 LL37 induces rapid phosphorylation of STAT3 in HaCaT cells, related to Figure 7. (A) WB analysis of p-STAT3 (Tyr705) and STAT3 in HaCaT cells after LL37 stimulation for the indicated durations (n = 3). Data are presented as mean ± SD; $^{*}P < 0.05$, $^{**}P < 0.01$, $^{***}P < 0.001$; ns, not significant.

**Gene symbol**	**Sequence**
GAPDH-human-F	GTCTCCTCTGACTTCAACAGCG
GAPDH-human-R	ACCACCCTGTTGCTGTAGCCAA
ACTA2-human-F	CTATGAGGGCTATGCCTTGCC
ACTA2-human-R	GCTCAGCAGTAGTAACGAAGGA
IL6-human-F	TACATCCTCGACGGCATCTC
IL6-human-R	TTCACCAGGCAAGTCTCCTC
IL1B-human-F	CAGTTGTTGTGGCCATGGAC
IL1B-human-R	GGAGCGTGCAGTTCAGTGAT
IL8-human-F	TTTTGCCAAGGAGTGCTAAAGA
IL8-human-R	AACCCTCTGCACCCAGTTTTC
CCL2-human-F	CAGCCAGATGCAATCAATGCC
CCL2-human-R	TGGAATCCTGAACCCACTTCT
CCL20-human-F	TGCTGTACCAAGAGTTTGCTC
CCL20-human-R	CGCACACAGACAACTTTTTCTTT
CXCL2-human-F	AGAAGGAAGGAGGAAGCTTATTG
CXCL2-human-R	CTGCAGCTGTGTCTCTCTTT

Table S1 Detailed primer sequence in this research, related to Figure 3 and Figure 7.

**Patient ID**	**Group**	**Subtype**	**Age**	**Gender**
A01	Rosacea	ETR	42	Female
A02	Rosacea	ETR	42	Female
A03	Rosacea	ETR	38	Female
A04	Rosacea	PPR	32	Female
A05	Rosacea	PPR	27	Female
A06	Rosacea	PPR	29	Male
A07	Rosacea	PPR	26	Female
A08	Rosacea	PPR	29	Male
A09	Rosacea	PPR	28	Male
A10	Rosacea	PPR	19	Female
A11	Rosacea	PPR	30	Female
A12	Rosacea	PPR	30	Female
A13	Rosacea	PPR	35	Female
A14	Rosacea	PPR	35	Female
A15	Rosacea	PPR	28	Female
A16	Rosacea	PPR	42	Female

A17	Rosacea	PPR	18	Female
A18	Rosacea	PPR	36	Female
B01	Control	—	26	Female
B02	Control	—	26	Female
B03	Control	—	37	Male
B04	Control	—	27	Female
B05	Control	—	30	Female
B06	Control	—	25	Male
B07	Control	—	26	Male
B08	Control	—	27	Female
B09	Control	—	31	Female
B10	Control	—	32	Female
B11	Control	—	30	Male
B12	Control	—	19	Male
B13	Control	—	31	Female
B14	Control	—	32	Female
B15	Control	—	27	Male
B16	Control	—	33	Female
B17	Control	—	27	Male
B18	Control	—	25	Female
B19	Control	—	25	Female

Table S2 Patient characteristics and demographics, related to Figure 1.
